# The Role of Small Molecules and Their Effect on the Molecular Mechanisms of Early Retinal Organoid Development

**DOI:** 10.3390/ijms22137081

**Published:** 2021-06-30

**Authors:** Ellie L. Wagstaff, Andrea Heredero Berzal, Camiel J. F. Boon, Peter M. J. Quinn, Anneloor L. M. A. ten Asbroek, Arthur A. Bergen

**Affiliations:** 1 Department of Human Genetics, Amsterdam UMC, University of Amsterdam (UvA), 1105 AZ Amsterdam, The Netherlands; a.l.tenasbroek@amsterdamumc.nl; 2 Department of Ophthalmology, Amsterdam UMC, University of Amsterdam (UvA), 1105 AZ Amsterdam, The Netherlands; a.herederoberzal@amsterdamumc.nl (A.H.B.); Camiel.boon@amsterdamumc.nl (C.J.F.B.); 3 Department of Ophthalmology, Leiden University Medical Center (LUMC), 2333 ZA Leiden, The Netherlands; 4 Jonas Children’s Vision Care and Bernard & Shirlee Brown Glaucoma Laboratory, Columbia Stem Cell Initiative, Departments of Ophthalmology, Pathology & Cell Biology, Institute of Human Nutrition, Vagelos College of Physicians and Surgeons, Columbia University, New York, NY, USA; Edward S. Harkness Eye Institute, Department of Ophthalmology, Columbia University Irving Medical Center—New York-Presbyterian Hospital, New York, NY 10032, USA; pq2138@cumc.columbia.edu; 5 Netherlands Institute for Neuroscience (NIN-KNAW), 1105 BA Amsterdam, The Netherlands

**Keywords:** retinal organoids, retinogenesis, cell signaling, human development, disease modeling, stem cells

## Abstract

Early in vivo embryonic retinal development is a well-documented and evolutionary conserved process. The specification towards eye development is temporally controlled by consecutive activation or inhibition of multiple key signaling pathways, such as the Wnt and hedgehog signaling pathways. Recently, with the use of retinal organoids, researchers aim to manipulate these pathways to achieve better human representative models for retinal development and disease. To achieve this, a plethora of different small molecules and signaling factors have been used at various time points and concentrations in retinal organoid differentiations, with varying success. Additions differ from protocol to protocol, but their usefulness or efficiency has not yet been systematically reviewed. Interestingly, many of these small molecules affect the same and/or multiple pathways, leading to reduced reproducibility and high variability between studies. In this review, we make an inventory of the key signaling pathways involved in early retinogenesis and their effect on the development of the early retina in vitro. Further, we provide a comprehensive overview of the small molecules and signaling factors that are added to retinal organoid differentiation protocols, documenting the molecular and functional effects of these additions. Lastly, we comparatively evaluate several of these factors using our established retinal organoid methodology.

## 1. Introduction

### Development of the Retina

**Figure 1:**
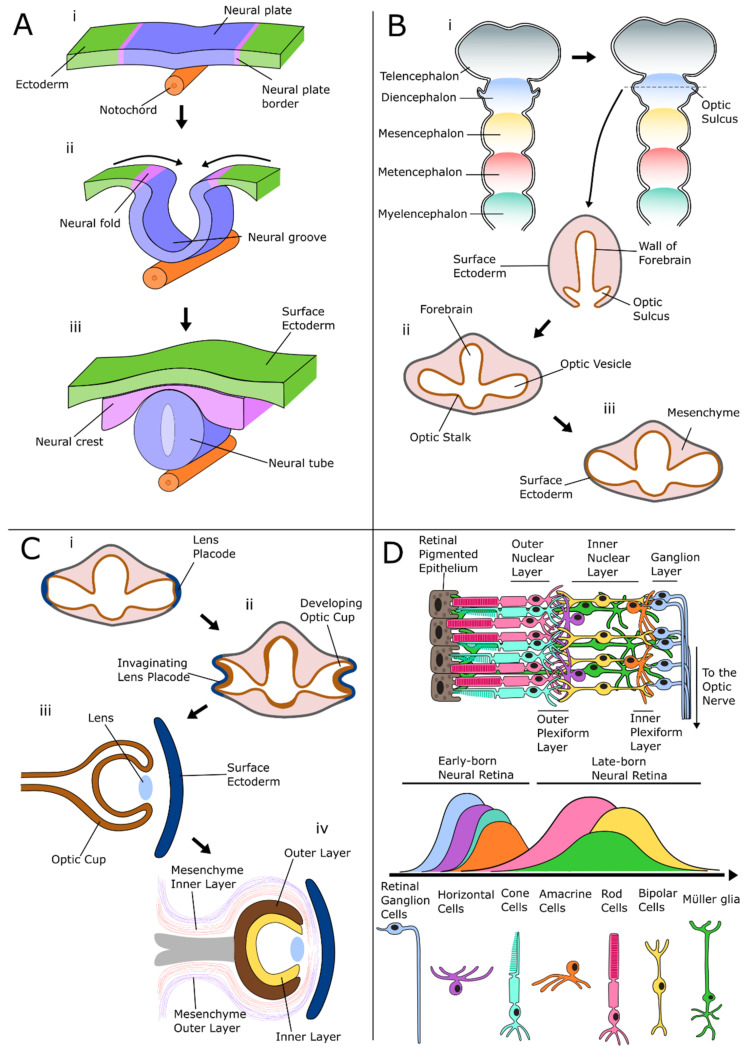
The phases of the embryological development of the eye. (**A**) The first stage of development is the formation of the neural tube. (**Ai**) The notochord stimulates the neural plate to be drawn inwards from the ectoderm. (**Aii**,**Aiii**) The neural folds meet and fuse, creating the neural tube and neural crest. (**B**) The formation of the optic vesicle. (**Bi**) The neural tube develops into five secondary vesicles. (**Bii**) The optic sulcus grows out laterally from the diencephalon, enlarging the distal area to form the optic vesicle and pinching the proximal area to form the optic stalk. (**Biii**) The optic sulcus continues to grow until it reaches the surface ectoderm. (**C**) Development of the major eye structures. (**Ci**) The area of the surface ectoderm touching the optic vesicle thickens and forms the lens placode. (**Cii**) The lens placode then invaginates, before pinching off from the surface ectoderm to become the lens. (**Ciii**) This invagination results in a double-layered optic cup structure. (**Civ**) The outer layer later becomes the retinal pigmented epithelium, whereas the inner layer develops into the neural retina. The surrounding mesenchyme helps form structures such as the cornea, choroid, and ciliary body. (**D**) The retina is a laminated structure consisting of many different cells, which can be split into early-born neurons and late-born neurons. The early-born neurons include retinal ganglion cells, horizontal cells, cone cells and amacrine cells, whereas the late-born neurons consist of the rod cells, bipolar cells, and Müller glial cells. These are interconnected in the different layers of the retina, including the outer nuclear layer, the outer plexiform layer, the inner nuclear layer, the inner plexiform layer, and the ganglion layer.

Human eye development can be separated into four main stages: the development of the neural tube, the formation of the optic vesicle, the invagination of the double layered optic cup, and the development of the fully differentiated retina. First, neural tube formation is induced by the developing notochord, a long rod that forms along the anteroposterior axis of the embryo (Figure 1A). The notochord secretes growth factors that prompts the differentiation of the overlying ectoderm into the neural ectoderm via hedgehog, BMP and Wnt signaling [1]. Subsequently, this structure thickens into the neural plate (Figure 1Ai). The lateral edges of the neural plate then rise to form neural folds, fusing to form the neural tube, which is the precursor to the brain, eye, and spinal cord (Figure 1Aiii). Neural tube formation is known as primary neurulation and occurs by the end of the fourth week of embryonic development.

The second major stage towards eye development can be considered as the formation of the optic vesicle. Once the neural tube has been formed, five secondary vesicles appear at the rostral part of the tube. These are the telencephalon, diencephalon, mesencephalon, metencephalon, and the myelencephalon, which give rise to the forebrain (telencephalon and diencephalon), midbrain (mesencephalon), and hindbrain (metencephalon and myelencephalon). Next, the area at the base of the diencephalon, on the border with the mesencephalon, forms a thickened area on either side (Figure 1Bi), which is the first sign of the bilateral separation of the optic tissue [2]. As the area continues to thicken and grow, it bulges and forms the optic sulci at embryonic day 22 (E22) [2,3]. Further in the development process, the distal area of the sulcus enlarges to form an optic vesicle at E24, whereas the proximal area restricts and forms the optic stalk (Figure 1Bii). The optic vesicle continues to grow laterally until it meets the outer surface ectoderm layer, which still surrounds the neural tube (Figure 1Biii) [4].

The third stage of eye development is the invagination of the optic vesicle and subsequent development of the other major eye structures (Figure 1C). Once the optic vesicle meets the outer surface ectoderm layer, the area of the surface ectoderm that overlays the optic vesicle thickens and form the lens placode (Figure 1Ci). The lens placode continues to thicken and move inwards towards the optic vesicle, pinching in to become the lens pit. As the lens pit forms, the optic vesicle also starts to invaginate into a double layered structure known as the optic cup by E32 (Figure 1Cii) [5]. The lens pit detaches from the surface ectoderm, becoming a separate structure that ultimately develops into the lens (Figure 1Ciii). The future choroid and sclera are formed by the mesenchyme, which surrounds the neural tube and optic vesicle throughout development [6]. The mesenchyme exists as two layers: an outer fibrous layer and an inner vascular layer. In eye development, the outer fibrous layer immediately touches the surface ectoderm where the lens placode was once located to form the cornea, along with the current surface ectoderm. In the posterior section, which surrounds the developing retina, the fibrous layer forms the sclera, whereas the inner vascular layer forms the choroid and part of the ciliary body (Figure 1Civ).

Finally, once the outer eye structure has formed, the retina can develop. Two parts of the retina will develop: the retinal pigment epithelium (RPE) and the neural retina (Figure 1D). The neural retina is formed from the inner wall of the optic cup which proliferates and differentiates, forming its multilayered structure. The outer wall remains as a single cuboidal layer which becomes the RPE [7]. The neural retinas go through an early phase and a late phase of development, with different types of cells differentiating and maturing in subsequent waves [8]. The first phase is characterized by the generation of ganglion cells, horizontal cells, cone photoreceptor cells and amacrine cells (Figure 1D). Retinal ganglion cells (RGCs) sit on the innermost layer of the retina and through their topographically mapped axonal projections transmit electrical signals to the brain [9]. Horizontal cells help integrate and regulate the input from multiple photoreceptors and localize to the outer plexus layer of the retina. Cone photoreceptors are located on the outermost layer of the retina and are responsible for distinguishing between colors under normal lighting conditions but cannot perform in areas with dim light. Although they are generally outnumbered in the retina by the rod photoreceptors, they converge in an area of the neural retina called the fovea. Here, there are no rods present, and this area gives the best visual acuity of the eye. Amacrine cells are the last of the early-born retinal neurons and operate within the inner plexiform layer. Here, they interact with retinal ganglion cells and bipolar cells, affecting the output of the bipolar cells. The late phase of retinogenesis is characterized by the development of rod photoreceptors, Müller glial cells, and bipolar cells. Rod photoreceptors work in tandem with cone photoreceptors, allowing vision in dim light, but do not distinguish color. On the outermost layer of the retina, rods outnumber cones, by roughly 20:1 [10]. Müller glial cells are support cells to the other neural retinal cells. Whilst their cell bodies are located in the inner plexiform layer, Müller glial cell processes span the entire retina, from the inner limiting membrane to the outer limiting membrane. This helps maintain the laminar structure of the retina by providing stability. Müller glial cells also protect the retinal neurons by releasing neurotrophic factors, and maintaining the metabolic and electrophysiological homeostasis of the retina [11]. Bipolar cells are tasked with relaying the electrical stimulus from the photoreceptors to the retinal ganglion cells. Spanning across from the outer plexiform layer into the inner plexiform layer, bipolar cells can be separated into two major groups: Off-bipolar cells and On-bipolar cells. Off-bipolar cells continuously fire in the dark and are suppressed by light, while On-bipolar cells are excited by light and suppressed in the dark.

## 2. The Signaling Pathways of the Developing Retina

In this section, we describe the major signaling pathways involved in the development of the human eye: hedgehog (Section 2.1), BMP (Section 2.2) and Wnt (Section 2.3) signaling all have important functions in the early differentiation of the neural tube. TGF-β (Section 2.2) signaling helps protect retinal neurons from apoptosis during early development, and notch (Section 2.4) signaling has a key role in retinal progenitor cell development and the production of distinct retinal populations. We provide an overview of each signaling pathway, with essential steps which can be targeted by small molecule additions.

### 2.1. Hedgehog Signaling

**Figure 2:**
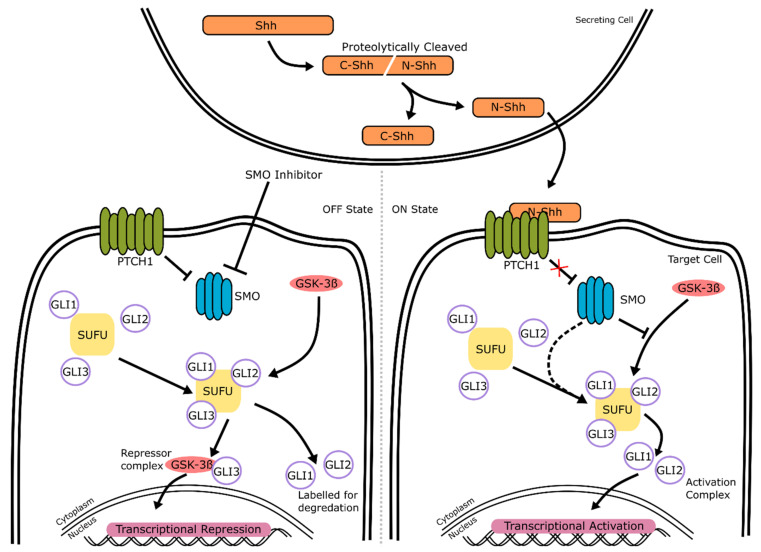
The hedgehog signaling pathway. (**Top**—Stage 1): to initiate hedgehog signaling, the secreting cell cleaves the Shh protein into two domains: N-Shh and C-Shh. N-Shh is then secreted into the extracellular domain. (**Left**—Stage 2): cells that have not yet been targeted by Shh exist in an “OFF State”. In this state, PTCH1 inhibits Smo, which allows SUFU to bind the Gli proteins, keeping them in an inactive state. Gli3 forms a repressor complex with GSK-3β, whilst Gli1 and Gli2 are marked for degradation. The repressor complex translocates to the nucleus, where it represses gene transcription. (**Right**—Stage 3): once the N-Shh has bound to the transmembrane protein PTCH1, cells transition into an “ON State”. In this state, PTCH1 no longer inhibits Smo, which in turn stops SUFU-mediated inactivation of the Gli proteins. This impedes the formation of the repressor complex and allows Gli1 and Gli2 to form an activation complex, which translocates to the nucleus and activates the transcription of target genes.

The hedgehog signaling pathway is presented in summary in Figure 2. It is an integral part of human embryonic development and is tasked with ensuring the proper differentiation of embryonic cells. It also initiates the formation of the neural tube after hedgehog signaling initiating factors are secreted by the notochord. Hedgehog signaling is conserved among many species. Vertebrate hedgehog homologues consist of three classes: Desert hedgehog, (Dhh), Indian hedgehog (Ihh), and Sonic hedgehog (Shh). The most well-understood class is Sonic hedgehog, which is involved in the regulation of the central nervous system and induction of motor neuron differentiation [12,13], although all three activate the hedgehog signaling pathway.

#### 2.1.1. Hedgehog Signaling: Mechanism of Action

The process of hedgehog signaling in vertebrates is complex and relies on concentration gradient-based cell to cell signaling. The pathway works through a multistep process. There are cells that secrete the ligand, a protein known as Sonic hedgehog (Shh), and target cells that relay the signal cascade to activate target gene transcription (Figure 2). A location-dependent spatial Shh concentration gradient between secreting cells and target cells determines the mode of action of the signaling pathway. Target cells located close to the secreting cells will receive higher Shh doses than cells further away, and develop into different cell types, which subsequently influences embryonic patterning and brain organization.

The signaling process can be divided into three stages: firstly, secreting cells release the Shh protein into the extracellular matrix. Secondly, the Shh protein binds to a receptor on a target cell that has not yet been targeted by Shh and is therefore in an “OFF State”. Thirdly, the target cell becomes activated by Shh and turns into an “ON State”, thus activating the transcription of target genes. These stages are described in detail below. The first stage is characterized by secreting cells that cleave the Shh protein into two parts, N-terminal Shh (N-Shh), and C-terminal Shh (C-Shh) (Figure 2, top). These are released into the extracellular matrix. The second stage is initiated when N-Shh binds to PTCH1 on the target cell. A Shh target cell is, without signaling occurring, in the “OFF state” (Figure 2, left): the transmembrane protein (and receptor of Shh) PTCH1, inhibits Smoothened (Smo), a signal transducer. Smo inhibition allows suppressor of fused (SUFU) to bind to and inactivate the Gli proteins. This activates the transcriptional repressor complex of Gli3 and Glycogen synthase kinase -3β (GSK-3β), whilst marking Gli1 and Gli2 for degradation, causing a transcriptional repression of hedgehog target genes. The inactivation of Smo, for example, by the selective Smo inhibitor cyclopamine, can cause this loss of expression even in the absence of PTCH1 [14]. The third stage is characterized by the transformation of the target cell into an “ON State” after the binding of N-Shh to PTCH1 (Figure 2, right): the binding of Shh to PTCH1 prevents the repression of Smo. This allows Smo to inhibit both the inactivation of the Gli proteins by SUFU, and the binding of GSK-3β to Gli3. This activates the transduction pathway, whereby the Gli1 and Gli2 transcription factors are activated and translocate to the nucleus, where they control the transcription of target genes, such as *PAX2* and *OTX2* [15,16,17]. The molecular mechanism by which Smo and Gli activates target gene expression is not fully known, and it has been shown that PTCH1 can also repress target gene transcription through a process independent of Smo [18].

#### 2.1.2. Hedgehog Signaling: Neural and Retinal Development

In neural development, the eye field develops from an area of the anterior neural plate that folds into a structure that will become the neural tube. This is stimulated by hedgehog signals secreted from the notochord, which initiates the invagination of the neural plate along the midline. Shh also has an important role in the bilateralization of the neural plate that results in determination of the eye fields, whereby cells acquire different identities depending on their relative spatial position within the eye. Studies in Shh knockout mice showed defects in bilateralization, and mice developed cyclopia [19].

Once the bilateral separation occurs, Shh is still required in the development and formation of the mature eye and specific cell types. Indeed, hedgehog signaling is intrinsically involved in all stages of retinal ganglion cell development. Neumann and Nuesslein-Volhard (2000) showed in zebrafish models that a wave of Shh signaling moves throughout the eye, preceding an expressional wave of *atonal*, a known gene involved in early retinal ganglion cell differentiation [20]. Indeed, further experiments in zebrafish models demonstrated that, after initial ganglion cell development, hedgehog signaling was involved in the guidance of retinal ganglion cells axons exiting the eye through the optic nerve. Shh (and Smo) mutations in zebrafish models resulted in abnormal optic nerve growth, and some axons even failed to exit the eye [21]. Furthermore, continued signaling is needed for the maturation of the optic cup, and the inactivation of Shh at this stage leads to a hypoplastic optic nerve in murine models [22]. Finally, Shh signaling might also be involved in the guidance of retinal ganglion cell axons in the optic chiasm through the establishment of nerve fibers crossing along the rostral cranial axis. The addition of Shh causes axonal growth to slow in chick retina explants [23].

Shh signaling does not only affect the neural retina but is also involved in the differentiation and maturation of the RPE, which requires Shh signaling during the later stages of the development of the optic cup [24]. In zebrafish, Shh is temporally highly expressed in the RPE layer immediately before a wave of photoreceptor differentiation, with a reduction in Shh by antisense oligonucleotides inhibiting photoreceptor development [25]. In lens development, hedgehog signaling disrupts the differentiation of fiber cells in mouse models [26].

### 2.2. TGF-β/BMP Signaling

**Figure 3:**
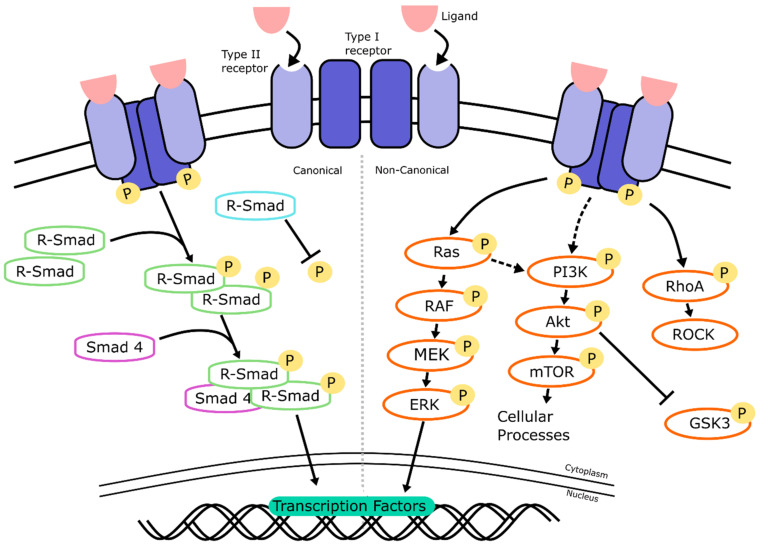
The TGF-β/BMP signaling pathway. TGF-β and BMP ligands bind to two type II receptors, which recruit two type I receptors, and a complex is formed. The type I receptors are phosphorylated (P), initiating both the canonical and non-canonical signaling pathways. (**Left**) In the canonical pathway, phosphorylation of the type I receptors results in the recruitment of two R-Smads. The two R-Smads are phosphorylated and recruit SMAD4, forming a complex which translocates to the nucleus, where it regulates gene transcription. I-Smads inhibit R-Smad phosphorylation. (**Right**) In the non-canonical pathway, Smad-independent interactions occur within different kinase cascades, such as the Ras-ERK-MAPK pathway, the PI3k-Akt-mTOR pathway and the RhoA pathway. This sequential recruitment and phosphorylation of downstream kinases also results in translocation to the nucleus, where transcription of TGF-β and BMP target genes is regulated, such as *Sp1*, Forkhead Box (FOX)-related genes, and basic helix–loop–helix (bHLH)-related genes.

The transforming growth factor-beta (TGF-β) signaling pathway is presented in Figure 3. It consists of a large superfamily of interacting proteins, growth factors, and Activins that govern many different cellular processes, from cell development to apoptosis. In humans, the TGF-β superfamily consists of over 30 known members that encode for the different TGF-β isoforms, bone morphogenetic proteins (BMPs), growth and differentiation factors (GDFs) and Activins [27]. All have differing roles in development and can be split into groups depending on which receptors they bind to. In retinal development, TGF-β signaling protects neurons from programmed cell death during development [28]. BMP proteins, along with GDFs, make up a large subset of the TGF-β superfamily [29]. The pathway is well conserved between species.

#### 2.2.1. TGF-β/BMP Signaling: Mechanism of Action

There are two major branches of this signaling pathway: (1) the TGF-β/Activin/Nodal pathway and (2) the BMP/GDF pathway [30]. Both of these branches work through canonical and non-canonical signaling (Figure 3).

**Figure 4:**
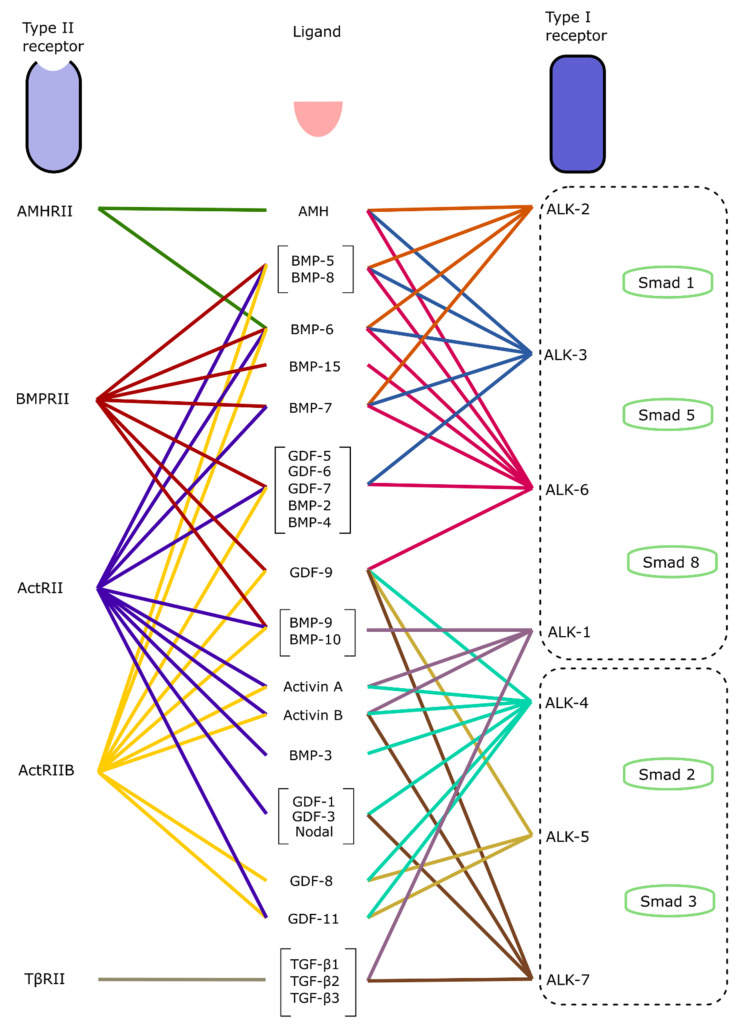
TGF-β and BMP signaling: ligand–receptor interactions involved in canonical and non-canonical signaling. TGF-β and BMP ligands can bind to many different combinations of type I and II receptors in both canonical and non-canonical signaling. There are five different type II receptors (**left**) which interact with the many different ligands (**center**). These ligand-bound type II receptors then form a complex with a corresponding type I receptor (**right**). However, in the canonical pathway, the specific type I receptor involved in the complex subsequently determines which R-Smad is recruited. ALK-1, -2, -3, and -6 receptors are commonly used in BMP signaling, and recruit SMAD 1, 5 and 8. The TGF-β branch more commonly works through type I receptors ALK-4, -5, and -7, that recruit SMAD 2 and 3.

The signaling cascade is initiated by the binding of a ligand to a type II cell membrane-bound TGF-β receptor, which then recruits a corresponding type I receptor depending on the original ligand (Figure 3). In humans, there are five type II receptors (TβRII, ActRII, ActRIIB, BMPRII. AMHRII) and seven type I receptors (ALK-1, -2, -3, -4, -5, -6, -7), which are activated by different TGF-β ligands [31] (Figure 4). TGF-β, Activin and Nodal ligands generally bind to type I receptors ALK-4, -5, and -7. For example, TGF-β1, 2, and 3 all bind to the receptor type I ALK-1 and ALK-7, and the receptor type II TβRII. It is important to understand the specificity of ligands and their target receptors, as pathway agonists or small molecule inhibitors will only target certain receptors, such as SB431542, which is a selective inhibitor of ALK-4, ALK-5, and ALK-7 [32]. BMP ligands work through the type I receptors ALK-1, -2, -3 and -6. For example, BMP-5, -8a, and -8b bind to type I receptors -2, -3, and -6, and type II receptors ActRII, ActRIIB, and BMPRII.

Once activated, the signaling cascade can work in a SMAD-dependent (canonical) or SMAD-independent (non-canonical) manner. In the canonical branch of the pathway (Figure 3, left), the binding of a ligand to its receptor results in three subsequent actions: the formation of a type I/II membrane-bound receptor complex, the recruitment of specific R-SMADs, and the formation of R-SMAD/SMAD4 complexes that can enter the nucleus. These actions are next described in more detail: once a ligand binds, a complex of two type I and two type II receptors forms, which triggers the phosphorylation of the type I receptor. Depending on the ligand type, a specific SMAD protein is recruited and phosphorylated (Figure 4). Subsequently, intra-cellular SMAD–protein complexes form that can translocate to the nucleus, and they regulate gene expression. In general, SMADs can be separated into three groups: Receptor-regulated Smads (R-Smads), Common-Smads (Co-Smads), and Inhibitory Smads (I-Smads). R-Smads are the initial proteins that become phosphorylated after the type I/II receptor complex forms, depending on if TGF-β or BMP signaling is occurring. SMAD2 and SMAD3 are phosphorylated during TGF-β signaling after the recruitment of type I receptors ALK-4, -5, or -7, while SMAD1, SMAD5, and SMAD8 are targeted by the BMP branch after the recruitment of type I receptors ALK-1, -2, -3, or -6 (Figure 4) [33]. Once activated, these R-Smads bind to SMAD4, the only known co-Smad, and form trimers that translocate into the nucleus. The remaining SMAD6 and SMAD7 are classed as I-Smads, that bind to the R-Smads and inhibit the signaling cascade.

In the non-canonical branch (Figure 3, right), the cascade works through a Smad-independent manner via at least three signaling cascades, namely the extracellular signal-regulated kinase (ERK) (also known as mitogen-activated protein kinase (MAPK)), phosphatidylinositol-3 kinase (PI3K), and RhoA kinase cascades [34]. These three cascades can act independently or interact with each other to regulate gene transcription. In ERK signaling, for example (Figure 3, Ras-ERK), the cascade is initiated by a GTPase known as Ras, which is phosphorylated in response to the activation of the type I/II receptor complex formation. This results in a conformational change of Ras, allowing it to activate RAF, which in turn phosphorylates MEK and activates it. MEK finally phosphorylates ERK, also known as MAPK, which then directly activates or inhibits transcription factors such as *PAX6*, a key transcription factor essential for the development of the early brain and eye [35]. The kinase pathways involved in non-canonical signaling interact closely with each other, with Ras also activating the PI3K-Akt signaling pathway (Figure 3, PI3K-mTOR) [36]. The activated protein kinase Akt is involved in many different processes, depending on its downstream target. Frequently, Akt inactivates GSK-3 through phosphorylation [37].

#### 2.2.2. TGF-β/BMP Signaling: Neural and Retinal Development

In this section, we describe the role of TGF-β/BMP signaling in neural and retinal development. Throughout development, TGF-β and BMP signaling are involved in differentiation, proliferation and programmed cell death, which are discussed in this context below.

TGF-β signaling has an important role in programmed cell death during retinal development. The analysis of developing chick retinas showed the presence of the type II receptor TβRII, as well as the ligands TGF-β2 and TGF-β3 in the central retina and optic nerve head during an early period of programmed cell death [38]. Furthermore, TβRII deletion in mice showed a significant increase in the apoptosis of retinal neurons during the development of the embryo, resulting in fewer retinal neurons and functional abnormalities [28]. The activation of the ERK and PI3K-Akt pathways through insulin also acts as a survival factor in the early chick embryonic retina to combat apoptosis, when progenitors and ganglion cells are developing [39]. Once the early retina has formed, TGF-β signaling also affects the development and differentiation of specific cell types. Kim et al. (2005) showed that the binding of the ligand GDF11 to the ALK-4 and -5 receptors (Figure 4), controls the proliferation of retinal progenitor cells that express the early RGC-specific marker *ATOH7*, thereby limiting RGC development. GDF11 knockout mice showed significantly increased levels of RGCs, with around 50% more cells in the ganglion cell layer than wild type mice. The same authors showed that GDF15 has the opposite effect to GDF11 and promotes RGC differentiation in mouse retinal progenitor cells by suppressing GDF11-induced SMAD2 phosphorylation [40]. This was subsequently confirmed in human embryonic stem cell models, where SMAD2 inhibition by SB431542, a synthetic inhibitor of the type I receptors ALK-4, -5, and -7, increased RGC differentiation. Finally, in human models, the addition of recombinant GDF11 significantly reduced the expression of the RGC marker *POU4F1*, which indicates that GDF11 negatively regulates RGC development [41].

BMP signaling is a vital part of the development of the neural crest. One of the earliest events in neural crest development is the formation of the neural plate border, which forms in an area of the neural plate with less BMP activity [42]. The neural plate eventually develops into the optic vesicles in the early stages of retinogenesis (Figure 1). This has also been shown in chick models, where the inhibition of BMP alongside TGF-β affected neural tube dorsal–ventral patterning [43]. Building on this, dual SMAD inhibition has been successfully adapted to neuronal and retinal development from human stem cells [44,45,46]. BMP is involved in the early development of the eye, with murine models showing that BMP-2, -4, and -7 are highly present in the embryonic retina, before being downregulated in adult retinas. BMP4 especially has a significant role in this early period of development, as it stimulates progenitor cells to differentiate into retinal ganglion cells rather than other neural cells such as astrocytes [47]. BMP4 treatment has since been used regularly in retinal organoid cultures to induce retinal lineage development [48,49]. In zebrafish models, BMP signaling is necessary for the induction of photoreceptor differentiation through interaction with notch signaling [50]. Furthermore, the inhibition of BMP signaling in mice results in a reduction in the Müller glial cell-specific genes *Rlbp1* and *Glul*, showing the role BMP signaling plays in the development of retinal neurons [51]. In addition to roles in the differentiation of the retina, BMP signaling is involved in the development of other eye structures. BMP-7 null mutant mice were observed to have a range of eye defects, from abnormal lens development to the absence of the whole lens, retina and cornea [52]. Ras-ERK signaling (Figure 3, Ras-ERK) can also affect lens development. ERK signaling negatively regulates L-Maf, an important factor needed to promote the differentiation of lens cells from the neural retina [53]. GSK-3 plays an important role in neural and retinal development [54,55] and is also involved with a variety of other signaling pathways (Wnt, hedgehog, insulin and notch) [56,57,58,59]. Inhibited by Akt during non-canonical signaling, Marchena et al. also found that inhibition of GSK-3 by treatment with small molecule inhibitors led to retinal cell neuroprotection in a retinitis pigmentosa model [60].

### 2.3. Wnt Signaling

**Figure 5:**
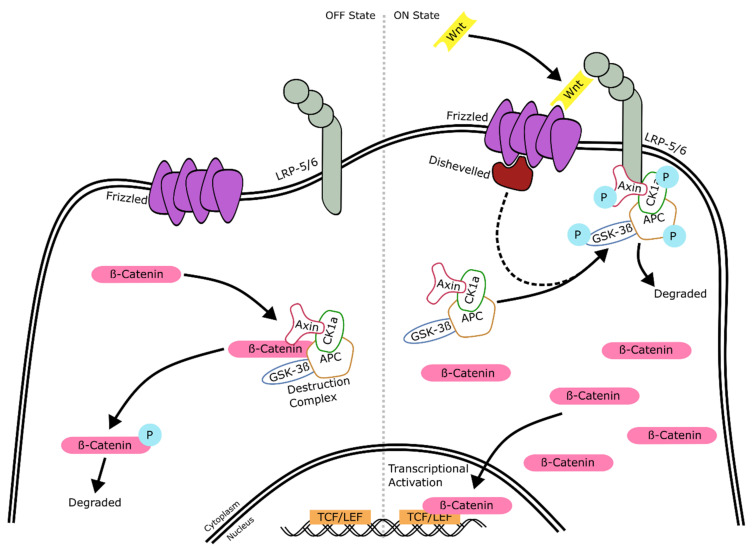
The canonical Wnt signaling pathway. (**Left**) Cells not activated by Wnt signaling exist in an “OFF state”. In the “OFF State” a so-called destruction complex is formed, comprised of Axin, CK1α, APC and GSK-3β. The complex phosphorylates (P) β-Catenin, which leads to its degradation. (**Right**) Wnt binds to the cell membrane receptor Frizzled (Fz), which turns the cell into an “ON State”. Frizzled then forms a complex with LRP-5/-6, which results in the recruitment of Disheveled (Dsh), a phosphoprotein. Dsh interacts with the destruction complex and translocates it to the membrane. Here, Axin binds to LRP-5/-6 and the destruction complex is degraded. This allows β-Catenin to accumulate in the cytoplasm and subsequently enter the nucleus, where it activates transcription of the TCF/LEF.

The Wnt signaling pathway is also highly conserved amongst species, and is responsible for regulating cell fate determination, cell migration and neural patterning. It operates through canonical (outlined in Figure 5) and non-canonical (not shown) signaling, similarly to the TGF-β/BMP pathway. The canonical pathway is the most understood and revolves around the β-catenin protein. The non-canonical pathway is less understood and is mainly involved in tissue polarity and the regulation of intracellular calcium levels through either the non-canonical planar cell polarity (PCP) pathway or the non-canonical Wnt/calcium pathway. These pathways both work independently of β-catenin. Since relatively little is known about the non-canonical pathway, we focus here on the description of the canonical pathway below.

#### 2.3.1. Wnt Signaling: Mechanism of Action

In the absence of Wnt signaling, cells are in the so-called “OFF state” (Figure 5, left). The “OFF State” status of the cell results in the degradation of β-catenin and subsequent transcriptional repression. This occurs through the formation of a “destruction complex”, comprised of Axin, adenomatosis polyposis coli (APC), Casein kinase 1a (CK1a), and GSK-3β. The destruction complex phosphorylates β-catenin, which marks it for degradation and becomes ubiquitinated [61]. The binding of Wnt to the cell membrane receptor results in the cell being in an “ON state” (Figure 5, right), which ultimately allows transcriptional activation. Initially, Wnt proteins bind to the transmembrane receptor Frizzled (Fz), which subsequently forms a complex with lipoprotein receptor-related proteins 5 and 6 (LRP5/6), triggering the recruitment of the phosphoprotein Disheveled (Dsh). Dsh disrupts the destruction complex by translocating it to the membrane, where AXIN1 binds to LRP5/6 and the complex is phosphorylated and subsequently degraded. Once the destruction complex has been disassembled, β-catenin can accumulate and localize to the nucleus, where it acts as a coactivator of the T-cell factor/lymphoid enhancing factor (TCF/LEF) family of transcription factors, which are involved in cell fate decisions.

#### 2.3.2. Wnt Signaling: Neural and Retinal Development

Wnt signaling has multiple essential roles in neural development and differentiation, including neural migration, the generation of the neural tube, axonal growth, and synapse formation [62,63,64,65]. In the eye, Wnt has been shown to play a key part in initial eye formation. In *Xenopus* models, the temporal and spatial expression of the Wnt signaling receptor Fz3 during development is restricted to the anterior neural plate, an anatomical precursor to the eye field. Fz3 is expressed throughout the developing optic vesicle, and affects the expression of *PAX6*, the master switch of eye development [66,67].

The importance of Wnt signaling in retinal development has been shown across species. In zebrafish, the activation of the Wnt pathway by the interaction of the ligand WNT8B and a Frizzled receptor inhibits the specification of the eye field [68]. Further research in murine models corroborated the hypothesis that *WNT8B* is a suppressor of early eye formation: *WNT8B*, usually expressed in the forebrain, expanded its expression into the optic pits in a conditional-knockout mouse model of *Six3* (a known early eye field marker), inhibiting the formation of neural retina [69]. This has since been shown in human stem cell models, where a knockdown of *WNT8B* restored neural retina formation in a *Pax6* knockout stem cell model [70]. Taken together, these data suggest that inhibitory *WNT8B* signaling is reversed by *Six3* expression, a known early eye field marker essential for the development of the neural retina. For specific retinal cell subtypes, Wnt signaling is active during injury, exerting neuroprotective effects. Activation by Wnt3a protected immortalized rat RGC-5 cells from cell death when cultured in elevated pressure conditions [71]. Similarly, Wnt signaling protected photoreceptors in an inherited retinal degeneration mouse model, increasing the pro-survival protein Stat3 [72]. Finally, after laser-induced injuries to mice retinas, the activation of Wnt signaling led to an increase in the proliferation of Müller glial cells [73]. Wnt inhibition is also often paired with the simultaneous inhibition of SMAD in human stem cell research, although it has also been used on its own as a way of directing cells to an eye field fate [74]. Nonetheless, the dual inhibition of Wnt and SMAD has been shown to direct the differentiation of stem cells efficiently into neural crest cells [75,76] as well as stimulate the genesis and differentiation of specific early-born neural retinal cells, such as cone photoreceptors and retinal ganglion cells, from the neuroectoderm [46,77].

Wnt signaling does not only play a role in the formation of the neural retina, but also has been implicated in the formation of the RPE. It is present in the dorsal section of the optic vesicle, which develops into the RPE [78]. Finally, Wnt signaling has a large role in the development of the lens and the initial lens epithelium. Although Wnt signaling is not necessary for lens fate determination, Wnt signaling is required for proper lens formation [79] and it is thought to play a role in the correct alignment of lens fiber cells [80].

### 2.4. Notch Signaling

**Figure 6:**
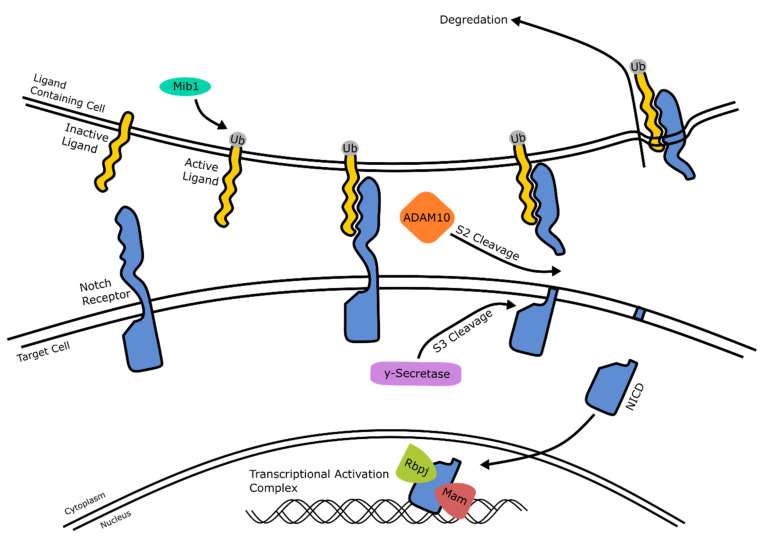
The notch signaling pathway. The multistep notch signaling pathway is presented from left to right in the figure. The first stage of notch signaling occurs in the ligand containing cell (**top**), where inactive ligands are ubiquitinated by Mib1 to become active. The ligands can then bind to a notch receptor present in the target cell membrane (**middle**). This receptor has an extracellular domain and an intracellular domain. The extracellular domain is cleaved by the metalloprotease ADAM10, before being endocytosed by the ligand containing cell. The intracellular domain in the target cell is then cleaved by γ-secretase and transported to the nucleus (**bottom**). Here, it forms a complex with Rbpj and Mam, activating transcription of target genes such as *HES1* and *HES5*.

Notch signaling, presented in Figure 6, refers to an intercellular signaling cascade invoked by cell–cell interaction. Highly conserved amongst species, it is responsible for multiple cell differentiation processes, including neural development, cardiovascular formation, and pancreatic cell specification [81,82,83].

#### 2.4.1. Notch Signaling: Mechanism of Action

In general, notch signaling occurs through a multi-step process (Figure 6, from left to right). First, the ligands located in the ligand-containing cell membrane are activated by the ubiquitin–protein ligase Mib1. There are two families of notch ligands: Jagged (Jag1 and Jag2) and Delta-like ligands (Dll1, DII3, Dll4) [84]. Next, this ligand binds to the notch transmembrane receptor on the target cell (four receptors are known: Notch1, Notch2, Notch3 or Notch4), where it forms a stable complex. After this interaction, the transmembrane receptor is cleaved on either side of the membrane of the target cell. The cleavage of the extracellular domain is known as S2 cleavage, and is initiated by the metalloprotease ADAM10 [85]. This cleavage triggers the notch intracellular domain (NICD) to be cleaved by γ-secretase, known as S3 cleavage. Finally, what remains of the extracellular ligand–receptor complex is endocytosed by the ligand-containing cell, while the cleaved intracellular domain of the receptor is transferred to the nucleus in the target cell. There, it forms a complex with recombinant binding protein j (Rbpj) and mastermind (Mam) that activates the transcription of several target genes, including *HES1* and *HES5* (described in detail below) [86].

#### 2.4.2. Notch Signaling: Neural and Retinal Development

In the developing central nervous system, notch plays an important role in the genesis, maintenance, and differentiation of neural progenitor cells. Chen et al. showed that notch inhibition leads to the accelerated differentiation of stem cells into the formation of neural rosettes by treating cultures with DAPT, a γ-secretase inhibitor [87]. Interestingly, these rosettes also possess Notch1, 2 and 3 receptors in their cell membrane, indicating that notch signaling has a role in maintaining neural cells in a progenitor state whilst inhibiting cell maturation [88].

Substantial experimental evidence points to notch signaling having a highly temporally defined role in the early formation of the eye. Indeed, Toonen et al. (2016) found that ADAM10 (Figure 6) conditional knockout mice showed an increased differentiation of early-born retinal neurons, which resulted in a highly disorganized retina lacking lamination [89]. Notch signaling affects the retinal progenitor cells in a similar fashion, with notch activation maintaining their progenitor state while inhibition leads to differentiation. However, continuous notch activation causes retinal progenitor cells to dedifferentiate and regain stem cell characteristics [90]. The knockdown of *Notch1* in early developmental stages in mice resulted in a smaller retina, likely due to the fewer numbers of retinal progenitor cells (RPCs). Furthermore, notch signaling is involved in the development of specific retinal neurons, such as ganglion cells, photoreceptors and Müller glial cells. Nelson et al. reported that there was a downregulation of notch signaling immediately preceding retinal ganglion cell development [91]. By following the expression of *Hes*, a family of well-known target genes of the notch signaling pathway that are found robustly in RPCs [92]; through reporter constructs, they found that while notch signaling is present in RPCs, it is not active in developing ganglion cells. This has also been shown in chick models, where antisense oligonucleotides reduced notch expression. This resulted in increased RGC production in vitro, and even reinitiated RGC development in vivo in retinas where ganglion development had stopped [93]. In human stem cells, the decreased activity of notch via the γ-secretase inhibitor DAPT also induced neural rosettes to develop into functional retinal ganglion cells [94]. Jadhav et al. (2006) and Mizeracka et al. (2013) showed that decreased notch signaling at an early stage leads to enhanced cone photoreceptor production, whereas a later knockdown increases the numbers of rod photoreceptors [95,96]. In retinal explant cultures, Mochizuki et al. (2014) found that Müller glial cell markers, such as *RLBP1* and *HEY2*, are significantly upregulated in the presence of notch signaling. In contrast, the inhibition of notch signaling leads to a decrease in glia precursor cells and fewer differentiated Müller glial cells [97].

## 3. Agonists and Antagonists Involved in Regulating the Signaling Pathways of the Developing Retina

To manipulate the aforementioned pathways in order to improve retinal differentiation, they can be controlled with known small molecules, proteins or transcription factors that either activate (agonists) or inhibit (antagonists) them. Below, we describe, pathway by pathway, several commonly used (ant-) agonists that affect the activity of signaling pathways.

### 3.1. Agonists and Antagonists of the Hedgehog Signaling Pathway

The regulation of the hedgehog signaling pathway with (ant-) agonists frequently revolves around the G protein-coupled receptor Smo (Figure 2). The main antagonist of Smo used in in vitro retinal models is cyclopamine. Cyclopamine is a naturally occurring steroidal alkaloid that targets Smo and has been used in in vitro models to induce the inhibition of hedgehog signaling at an early stage of development, facilitating retinal ganglion cell differentiation [98]. To complicate matters, the small molecule Smoothened Agonist (SAG) activates hedgehog signaling by binding to Smo itself, and therefore upregulates the activity of the Gli1/2 activation complex and subsequent target gene expression [99]. Thus, the addition of SAG into cultures at a late stage of development assists in the generation of retinal cells [100,101,102]. Finally, it is relevant to note here that hedgehog signaling can also be activated by the addition of recombinant Shh into cultures [103], although this is not common practice and, by convention, many groups prefer to use SAG.

### 3.2. Agonists and Antagonists of the TGF- β/BMP Signaling Pathway

In this section, we discuss the effect of small molecules on the activation or inhibition of TGF-β and, subsequently, the BMP signaling pathways (Figure 3). The inhibition or activation of the TGF-β/BMP signaling pathway is temporally controlled in in vitro retinal development, with inhibition within the first few days or activation in the second week of differentiation, promoting retinal development. The TGF-β pathway is commonly inhibited early in development through treatment with either the small molecule SB431542, or a recombinant protein named COCO. SB431542 is a potent inhibitor of the TGF-β, Activin and Nodal pathways (Figure 3). This antagonist exerts its effect by blocking the ALK-4, -5, and -7 receptors (Figure 4) [104]. SB431542, by itself or when combined with other inhibitors of the BMP pathway, efficiently accelerates the differentiation into the neuronal lineage [44,105,106]. SB431542 is typically added to cultures within the first seven days of cultures (Table 1). It improves the development of specific retinal cell types, such as photoreceptors, both in 2D cultures and 3D retinal organoid models [107,108,109]. COCO is part of the Dan family of TGF-β antagonists [110], and is commonly used as an inhibitor of the BMP branch of the signaling pathway (Figure 3). However, Bates et al. showed in a *Xenopus* model that COCO also affects the TGF-β side of the pathway by inhibiting Activin and Nodal signaling, therefore controlling germ layer specification [111,112]. Finally, COCO enhances the efficiency of photoreceptor differentiation [77], when used in combination with insulin growth factor 1 (IGF1). The prolonged treatment of COCO favored cone differentiation over rods [113]. Taken together, this shows that, in the context of the TGF-β/BMP signaling pathway, COCO has both agonistic and antagonistic properties [114]. It is, however, not commonly used in the protocols to generate retinal organoids, as small molecule inhibitors with the same or similar action are less expensive and can be more stable.

The cell signaling activity of the BMP pathway (Figure 3) can be affected by many readily available agonists and antagonists such as noggin, dorsomorphin, and BMP4. Noggin is a natural antagonist of the BMP signaling pathway. Once secreted, it binds to other BMP ligands, preventing them from binding to the receptors. Noggin inhibits at least BMP2, BMP4, BMP5, BMP6, BMP7, BMP13, and BMP14 [115], amongst other BMP ligands such as GDF-5 and GDF-6 [116,117]. High doses of noggin result in the specification of retinal and diencephalic regions at the expense of telencephalic regions in the developing *Xenopus* [118]. In human stem cell models, noggin has been routinely used to promote differentiation towards a photoreceptor fate [119,120]. However, in recent years, small molecule inhibitors, such as the BMP pathway antagonist dorsomorphin, have been favored over peptide antagonists such as noggin. In general, small molecule inhibitors are less expensive, have a higher penetrating capacity, and are more stable. Finally, dorsomorphin has also a more drastic effect on neural differentiation when compared to noggin [121,122].

Dorsomorphin was the first known small molecule inhibitor of the BMP pathway, and selectively inhibits the type I receptors ALK-2, -3, and -6 (Figure 4) in a dose-dependent manner. The inhibition of these receptors leads to downregulated gene transcription [123]. A derivative of dorsomorphin, named LDN193189, has a high affinity for ALK-2 and -3. Both dorsomorphin and LDN193189 efficiently block Smad-dependent and Smad-independent TGF-β pathways and inhibit BMP signaling [124]. However, it appears that dorsomorphin is a non-specific inhibitor of BMP and could potentially inhibit other kinases in vivo as well [125]. LDN193189 is thought to be a 100-times more potent inhibitor of the BMP pathway than dorsomorphin [126], but it too affects, less specifically, several other kinases. In retinal differentiation, both these small molecules have been used in organoid models to increase the efficiency of retinal generation [127,128]. However, they are usually used in combination with SB431542 as part of a dual SMAD inhibition to inhibit both the TGF-β and BMP sides of the pathway [107,108].

Retinal differentiation can be increased dramatically with the addition of an agonist, rather than an antagonist, of BMP signaling at a later time point. The addition of agonist BMP4 to retinal organoid cultures exposes the importance of timing in retinal development. Kuwahara et al. (2015) showed that treatment with BMP4 starting from day 6 onwards turned more than 95% of aggregates positive for *RAX*, a vital transcription factor essential for the development of the retina. Treatment with BMP4 starting from day 0, however, did not at all promote retinal or neural differentiation [127]. The timely addition of BMP4 between day 6 and 18 has since been adopted into many methods of generating retinal organoids [48,129,130,131].

### 3.3. Agonists and Antagonists of the Wnt Signaling Pathway

In this section, we describe the action of agonists and antagonists, such as CHIR99021 and IWR1e, on the activity of the Wnt signaling pathway. Wnt signaling, alongside TGF-β/BMP, is one of the most commonly used pathways to regulate retinal development in in vitro retinal models. It also temporally controls retinal development, with inhibition in the first two weeks promoting retinal development. In contrast, activation during the third and fourth week of differentiation also increases retinal development. The activation or inhibition of this pathway can be regulated to a multitude of readily available small molecules and proteins. Chen et al. (2009) previously identified a large group of chemically related inhibitors, named Inhibitors of Wnt Response (IWRs) [132]. The IWR1-*endo* (IWR1e) inhibitor has been the most frequently used IWR in retinal research. IWR1e regulates Wnt activation by stabilizing Axin, one of the constituents of the aforementioned β-catenin destruction complex (Figure 5). In organoid models, IWR1e is commonly used to promote the development of the retinal lineage and is most effectively added within the first 12 days of differentiation [74,133]. Similarly to IWRs, the small molecule XAV939 causes an accumulation of Axin in the cell, and decreases the amounts of β-catenin by promoting its degradation (Figure 5) [134], inhibiting Wnt signaling. Indeed, XAV939 treatment to early-stage stem cell-derived retinal cultures together with a BMP inhibitor resulted in an 84-fold increase in *PAX6* and 156-fold higher levels of *RAX* [135]. Yet another Wnt inhibitor used in retinal organoid development is the naturally occurring antagonist DKK1 protein, encoded by the *DKK1* gene. The protein has a high affinity to bind to LRP6 and prevents the formation of the Frizzled-LRP5/6 complex (Figure 5) [136]. This action subsequently prevents Disheveled from interacting with the destruction complex, thus inhibiting Wnt signaling. Given their same mode of action, treatment with XAV939 or DKK1 in vitro is interchangeable, and both biomolecules efficiently direct stem cells towards retinal progenitor and retinal ganglion cells [137,138,139]. Most research groups inhibit Wnt signaling in the early stages of in vitro retinal development. However, it has also been activated at later time points in retinal organoid development with the help of the agonist CHIR99021, an extremely potent GSK-3β inhibitor. GSK-3β plays a vital role in the inhibition of Wnt signaling, as it is an essential part of the destruction complex that ultimately labels β-catenin for degradation. CHIR99021 has also been found to specifically promote the differentiation of retinal progenitor cells into retinal pigmented epithelium that exhibited increased pigment and better morphology than non-treated controls [140].

### 3.4. Agonists and Antagonists of the Notch Signaling Pathway

**Figure 7:**
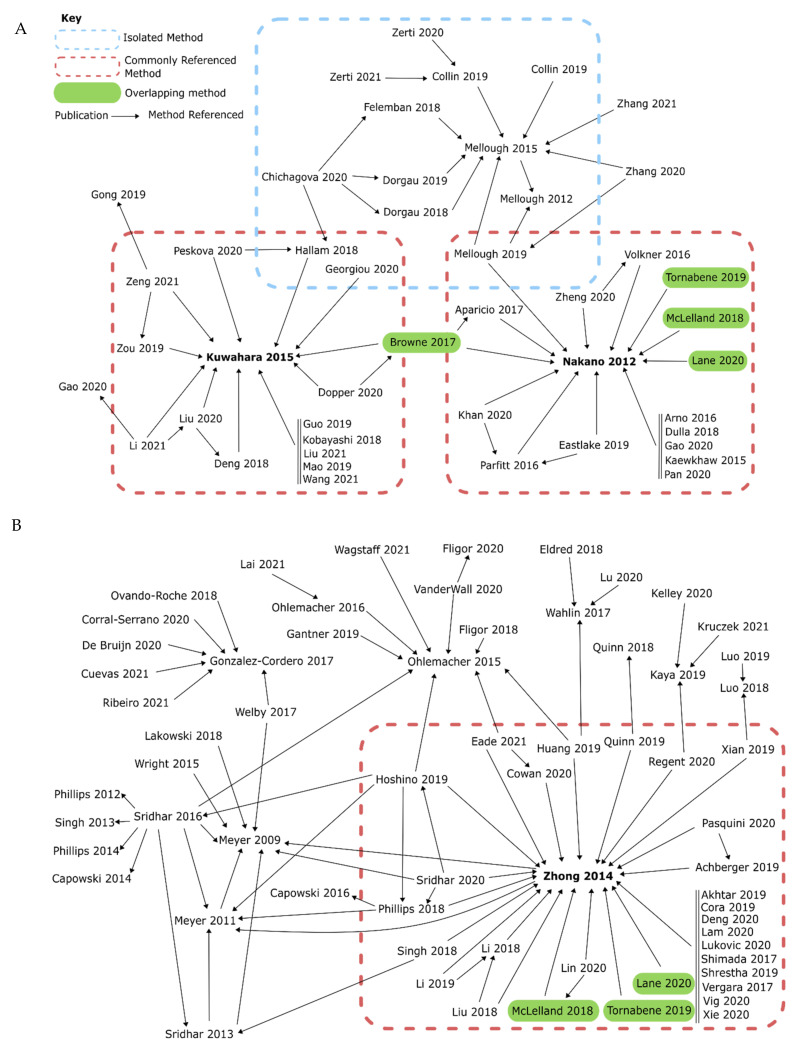

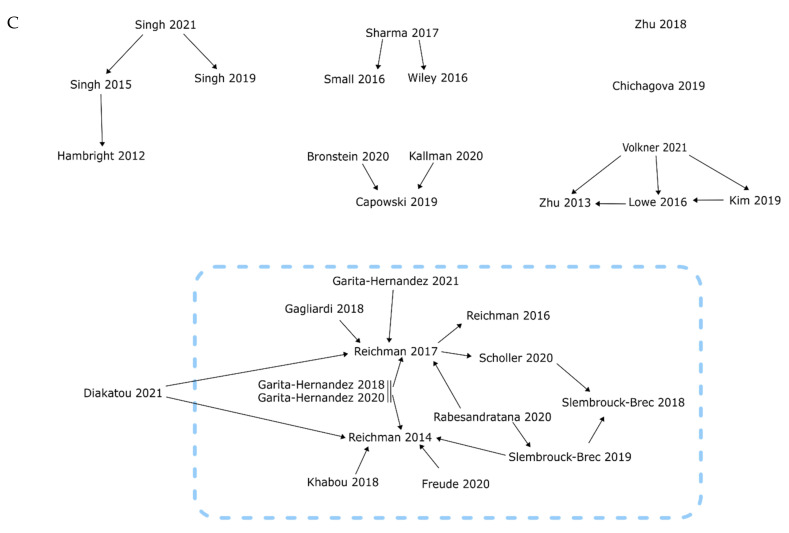
The evolution of organoid protocols. (**A**–**C**) Our literature search revealed that a majority of retinal organoid protocols had developed from three major papers described in Section 4.1 (highlighted in red boxes) before being adapted to fit individual needs. We also found that some protocols were isolated in specific groups (highlighted in blue boxes) but were still well-established within that group. There were also a handful of “stand-alone” protocols that had been rarely used. Some more recent methods combine multiple protocols, shown by multiple arrows or highlighted in green. (**A**) We found that many manuscripts were based on the methods of Kuwahara et al. [127] and Nakano et al. [154], leading to a high frequency of protocols generating embryoid bodies using single cells. On the other hand, some protocols were used mainly by a single group (highlighted in blue). (**B**) Zhong et al. [155] was the most commonly referenced method of generating retinal organoids, building upon the previous work of Meyer et al. [135,144], and outlines how this protocol, a method with relatively few steps or additional supplements, has been used as a base for many models, being adapted as needed. (**C**) Alongside stand-alone protocols used solely by a single group (in blue), there were also a small number of manuscripts that did not relate to other methods. All papers included either produced retinal organoids, or were referenced as a method of producing retinal organoids [48,49,74,101,107,108,109,113,127,129,130,131,135,143,144,145,146,148,149,150,152,154,155,156,157,158,159,160,161,162,163,164,165,166,167,168,169,170,171,172,173,174,175,176,177,178,179,180,181,182,183,184,185,186,187,188,189,190,191,192,193,194,195,196,197,198,199,200,201,202,203,204,205,206,207,208,209,210,211,212,213,214,215,216,217,218,219,220,221,222,223,224,225,226,227,228,229,230,231,232,233,234,235,236,237,238,239,240,241,242,243,244,245,246,247,248,249,250,251,252,253,254,255,256,257,258,259].

In in vitro retinal development, notch signaling (Figure 6) manipulation is predominately used to promote photoreceptor differentiation and maturation through the action of the small molecule DAPT, which is added at later stages of retinal development. DAPT is a γ-secretase inhibitor and blocks the S3 cleavage of the notch intracellular domain. This means that the NICD cannot form the transcriptional activation complex needed to regulate target gene expression. DAPT treatment results in the upregulation of cone and photoreceptor-specific genes, such as *CRX*. DAPT addition induces a shift from dividing progenitor cells to post-mitotic photoreceptor precursor cells [141,142]. Reichmann et al. (2014) found that a short week-long treatment of DAPT is sufficient to accelerate photoreceptor differentiation. Using their specific protocol (Figure 7C), they reported that DAPT treatment induced cell cycle exit in the majority of retinal progenitor cells, with around 40% of cells positive for *CRX*, a photoreceptor precursor marker [143].

### 3.5. Agonists and Antagonists Used in Retinal Models That Regulate Additional Signaling Pathways

Many other small molecules, proteins or transcription factors are frequently used to regulate retinal development. In this section, we discuss the effects of the small molecule SU5402, IGF1, and various isoforms of fibroblast growth factor (FGF) on retinal development.

The small molecule SU5402 is a potent and selective inhibitor of the ERK pathway, effecting the non-canonical branch of the TGF-β/BMP pathway (Figure 3). However, it is also heavily involved in the regulation of the fibroblast growth factor pathway (pathway not shown). When added on its own at early time points up until day 10 of differentiation, SU5402 treatment has led to a complete loss of *PAX6* and *RAX* expression, and consequently a loss of retinal fate [144]. In contrast, when added in combination with CHIR99021 at later stages of retinal development, SU5402 did promote retinal differentiation [145,146]. IGF1 has also been commonly added to retinal cultures over prolonged stages (Table 1) and plays a large role in both the development of the neural retina and long-term maturation of retinal ganglion cells [147,148]. Perhaps coincidentally, IGF1 is also one of the growth factors abundant in Matrigel that may accelerate retinal ganglion cell development in organoids models [149]. FGF signaling in retinal development is complex, as it depends on temporal and spatial signaling of a diverse group of FGF isoforms [144]. For example, FGF2 and FGF9 are constitutively expressed endogenously in the retina during development, and recombinant FGF2 can be added to cultures to favor the differentiation of the neural retina [150]. FGF9 expression is increased in optic vesicle stages of development, and it has been used in disease models involving optic vesicle malformation to (partially) rescue the phenotype [3,151].

## 4. Systematic Comparisons of Protocols and Supplements

### 4.1. Directly Comparable Uses of Agonists and Antagonists in Retinal Organoid Models

In this section, we describe a literature-based review of predominate methods used for retinal organoid generation. In general, these methodologies can be separated based on protocol origin (Figure 7A–C) or the timing and concentrations of external supplements used (Table 1), which we describe consecutively below. Finally, we included the results of a series of pilot experiments by which we experimentally compare the role of several key (ant-) agonists during early retinal organoid development using one of these established methods. The reason for describing these methodologies is that protocols used to generate retinal organoids vary greatly in-between laboratories, with different groups tending to have their own favored method.

**Figure 8:**
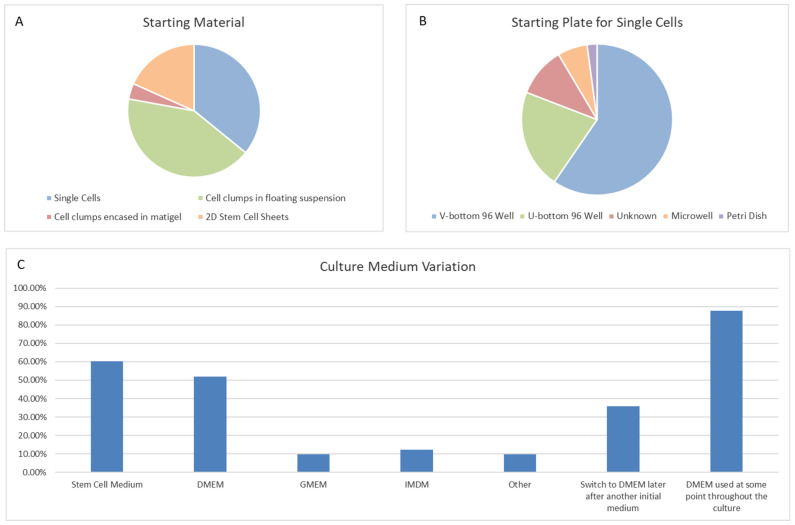
A breakdown of organoid techniques used. (**A**) Analysis of organoid protocols revealed that the most popular method of generating retinal organoids was starting with stem cell clumps in suspension, followed closely by single-cell aggregation. (**B**) For single-cell aggregation starts, the majority of methods used 96-well plates, with V-bottom plates being the most popular. (**C**) Different protocols use a variety of different media to start their differentiations, either alone or in combination. We found that starting with stem cell medium and/or DMEM was the most popular choice. Interestingly, although other protocols did start with other media such as GMEM or IMDM, we found that for long-term culturing, they switched to DMEM, meaning the overall number of protocols that use DMEM at some point in the differentiation is extremely high.

In general, embryoid bodies (EBs) can be generated in 3D floating culture, 3D Matrigel culture, or 2D adherent culture using simple reaggregation techniques or advanced microwell systems (Figure 8) [152,153]. The length of individual stages throughout the culture also varies between protocols and the progressive cell type-specific maturation depends on the focus of the protocol. Different methods of generating organoids also introduce a lot of variability that is protocol-dependent, such as the size and yield of organoids, and their ability to develop long-term lamination of the different cell layers. Protocols include many different small molecules or signaling factors, and nobody has systematically compared these “novel insights” in a reproducible manner using the same cell line and differentiation method. To understand which individual factors reported in the literature would work best to improve our previously published protocol, [149] with a 3D Matrigel-based start, we performed a systematic search of retinal organoid protocols. We made an inventory of which external factors were added at what time intervals in early retinal organoid development. We next chose the most commonly used conditions, compared them to our own (control) protocol and examined whether any differences in retinal organoid yield, morphology and gene expression were observed.

As cellular environments and development vary amongst species, we chose to concentrate solely on retinal organoids developed from human tissue. We identified 127 papers that generated retinal organoids (from 2011–2021) and reviewed their chosen methods. Although major upgrades and improvements have been published [152], we found that the majority of these methods could be traced back to three original major protocols [127,154,155] (Figure 7A,B). Nakano et al. (2012) used an initial Serum Free culture of Embryoid Body-like aggregates with Quick aggregation (SFEBq) method, with Rock Inhibitor (also known as Y-27632) and IWR1e present from day 0 to 12. Once an optic cup structure had started to form, they added SAG and CHIR99021 from day 15 to 18, before culturing for a longer term in standard medium with no more additions. Zhong et al. (2014) generated embryoid bodies using a 3D floating culture, with Blebbistatin present during the first day of culture (Table 1). No other factors are added other than taurine and retinoic acid, a pairing commonly used to enhance the long-term lamination of organoids. Finally, Kuwahara et al. (2015) also used an initial SFEBq culture to generate embryoid bodies, with Rock Inhibitor added from day 0 to 6. On day 6, BMP4 is added and gradually diluted with half medium changes up until day 18. Kuwahara et al. also examined the effects of other signal modulators on retinal development, including dorsomorphin, Wnt3a, IWR1e, SAG, cyclopamine, FGF2 and SU5402. However, they found BMP4 treatment to be most effective. Scientists are now exploiting the in vitro microenvironment of developing neuroepithelia through temporal manipulation with exogenous compounds to recapitulate the fetal microenvironment leading to enhanced retinal organoid differentiation. In addition, some groups have developed well-established protocols, which are isolated from the main cluster. For example, Goureau and coworkers used a 2D method of generating organoids by expanding stem cells until self-forming neuroretinal-like structures appear. These were then cultured in a 3D environment, with the transient treatment of FGF2 between day 28 and 35 [156,157,158].

Once we established which methods were previously used to generate retinal organoids, we systematically reviewed the steps of each protocol and indexed the external factors added to help the early development of the retinal lineage (Table 1), alongside other essentials, such as starting material and medium composition (Figure 8). We found that just over 40% of protocols use stem cell clumps in a suspension culture to generate embryoid bodies, as detailed in Zhong et al. [155] (Figure 8A). Around 35% of protocols use single cells to generate aggregates, which could be further sectioned into the type of plate used and the number of cells seeded per well. We found that the two most commonly used seeding densities and the most common plate type originated from two protocols: Nakano et al. [154], and Kuwahara et al. [127]. Nakano et al. generated aggregates using 9000 cells per well, and the popularity of the protocol is evident, with almost 30% of all single-cell aggregate methods using 9000 cells as their seeding density. Kuwahara et al. generated embryoid bodies using 12,000 cells per well, becoming the second most popular seeding density, with almost 25% of protocols using this amount. Both papers use V-bottom 96-well plates, resulting in 60% of methods with a single-cell start using these for their initial seeding (Figure 8B).

Most protocols used a starting medium of either a Dulbecco’s Modified Eagle Medium (DMEM)-based medium, a stem cell medium, or a defined ratio of the two (Figure 8C). We observed that approximately 30% of protocols used other types of media at the beginning of their cultures, such as Glasgow’s Minimal Essential Medium (GMEM) or Iscove’s Modified Dulbecco Medium (IMDM). Similar to the aforementioned seeding density or use of V-bottom plates, the use of GMEM or IMDM media could also be traced back to Nakano et al. and Kuwahara et al. No other methods incorporated either medium that were not based on the protocols of Nakano et al. or Kuwahara et al. (Figure S2). GMEM has been routinely used for mouse embryonic stem cell culture, and was present in previously published work from Nakano’s lab generating optic tissue from murine stem cells [260]. IMDM is a highly enriched medium that is well suited to highly proliferating cultures. Given the rapid expansion of cells in the early stages of generating aggregates, IMDM could be a favorable choice for use during the earlier stages of differentiation. Interestingly, however, we found that the majority of methods that did not start with a DMEM-based medium during the early differentiation stages often opted to switch to a DMEM base during later culture stages. This meant that a DMEM-based medium was used at some point throughout culturing in almost 90% of methods (Figure 8C). We also found that 95% of protocols included N2 and B27 supplements, either separately or together during the cultures. N2 supplement is used extensively in neural cultures and is often added in the earlier stages of retinal organoid differentiation. We here referred to the stage where stem cells are being directed down an anterior neural fate. Obviously, a neural fate stimulating supplement, such as N2, will help this process. Heparin was also commonly used in the first 3 weeks of differentiation alongside N2 supplement, as not only does heparin promote the proliferation of stem cells [261], but it can also induce neuronal differentiation [262]. Furthermore, it has been shown that heparin promotes Wnt signaling, which could also affect the differentiation of early retinal organoids [263,264]. Interestingly, we observed that at really late stages of differentiation, B27 supplement was replaced with N2 supplement for the remaining culture time. The reason for this could be because N2 supplement helps in the survival of post mitotic neurons. B27 supplement is used primarily to support the long-term viability of neuronal cells, and as such is commonly added during long-term culturing, after the initial differentiation into the neural lineage.

As we were concentrating on the initial development of the retina, we did not take into account supplements added for long-term development, such as taurine or retinoic acid [48,149,177,226]. Some factors influencing early development, such as Rock Inhibitor or Blebbistatin, were often used in cultures and were method independent. These can be used interchangeably and were mostly used in the first few days of cultures to initiate differentiation, reduce apoptosis and improve EB formation. However, Rock Inhibitor was frequently present for an extended time up to day 18, and is therefore also likely to affect the Rho-kinase section of the TGF-β non-canonical pathway outlined in Figure 3.

We also found that a selection of factors were added throughout development, whereas other additions were restricted to certain time points. For example, IWR1e, an inhibitor of the Wnt signaling pathway (Figure 5), was never added after day 14. In contrast, CHIR99021, a Wnt activator, was always added between days 14 and 24. This shows us that in stem cell-derived organoid models, the Wnt pathway needs to be regulated at specific early stages of retinal differentiation to initiate the proper development of the retinal lineage. In all the protocols reviewed, the BMP4 protein, simulating the BMP pathway (Figure 3), was always added before day 18, which hallmarks the beginning of developmental change from anterior neural fate to retinal fate. Note that, at this time point, neurospheres are still developing their anterior neural identity and have not yet developed into organoids. This reveals the essential role of the BMP pathway in the initial stages of development of neurospheres. The inhibition of BMP signaling was uncommon, with just 8 out of 131 protocols using noggin, dorsomorphin or LDN193189 inhibition for this purpose. Several other external factors, such as SU5402 or SAG, were frequently added in-between 12 to 24 days of early retinal organoid development, which is the time interval where many protocols transition from an anterior neural lineage to a full retinal lineage. Since SU5402 and SAG affect the FGF (not shown) and hedgehog (Figure 2) signaling pathways, respectively; their pathways appear to be essential for this transition in vivo, and studies have shown in chick explant models that SU5402 suppresses the differentiation of the early-born retinal ganglion cells [265]. DAPT, the γ-secretase inhibitor involved in notch signaling (Figure 6), is routinely added to improve photoreceptor differentiation and maturation [186,230,248]. In vitro, it was never added in the first 4 weeks of cultures, reinforcing that role of preparing the retina for late-born retinal neurons.

Other than temporal differences, we also observed that a range of concentrations was utilized for these aforementioned exogenous supplements. To complicate matters, some factors were kept at one constant concentration throughout methods used (SAG, FGF2), while for others (CHIR99021, SU5402), a whole range of concentrations were used, between 3 µM-3 mM and 2.5 µM-3 mM, respectively (Table 1). Interestingly, a number of frequently used supplement concentrations and/or timings related to individual groups or protocols, such as Blebbistatin, IWR1e or FGF2. Blebbistatin added from day 0 to 1 originated from Zhong et al. [155], which in part explains the popularity of these conditions. Similarly, IWR1e added at 3 µM from day 0 to 12 originated from the method of Nakano et al. [154], which has also been a heavily used method. In contrast, FGF2 supplements are routinely added almost solely by the group of Olivier Goureau [156] (Figure S2). Finally, we found that the dual SMAD inhibition of the TGF-β and BMP pathways by SB431542 and LDN193189 was an exclusive pairing and always used together.

**Table 1:** Summary of the additions used to promote retinal differentiation in organoid protocols and their frequency, concentration, and time interval of use. Analysis of the literature uncovered many supplements that are added to regulate signaling pathways throughout the development of retinal organoids. We decided to use the conditions and concentrations highlighted with an asterisk (*) and test their effect on retinal differentiation in our organoid protocol, representing the more popular time points or concentrations used for a range of conditions affecting different signaling pathways. For further explanation of the individual additions used, see text.

Description		Concentration	Length of Addition	Reference
BDNF	Activator of TGF-β and BMP signaling through PI3K and ERK signaling cascades	20 ng/mL	Day 63 onwards	Singh ‘19 [235]
bFGF	FGF signaling ligand	10 ng/mL	Day 14 onwards	Singh ‘15 [234], Singh ‘19 [235], Singh ‘21 [236]
Blebbistatin	Inhibitor of NMII-ATPase, downstream of Rock inhibition	5 µM	Day 0–1	Lu ‘20 [211]
		10 µM	Day 0–1	Achberger ‘19 [159], Akhtar ‘19 [160], Cora ‘19 [165], Deng ‘20 [170], Lane ‘20 [202], Li ‘19 [203], Luo ‘18 [214], Luo ‘19 [213], Quinn ‘18 [226], Quinn ‘19 [225], Tornabene ‘19 [240], Vergara ‘17 [242], Vig ‘20 [243], Xian ‘19 [250], Xie ‘20 [251], Zhong ‘14 [155]
		10 mM	Day 0–1	Li ‘18 [204], Lin ‘20 [206], Liu ‘18 [209]
BMP4	BMP signaling ligand	50 ng/mL	Day 6–15	VanderWall ‘20 [241]
			Day 6–18	Mao ‘19 [215]
		55 ng/mL	Day 6–15	Döpper ‘20 [108]
			Day 6–20	Khan ‘20 [196]
		100 ng/mL	Day 0–10	Meyer ‘09 [144]
		0.05 µg/µL	Day 5–20	Hoshino ‘19 [189]
		0.5 µg/µL	Day 6–12	Sridhar ‘20 [237]
		1.5 nM	Day 6–15	Bronstein ‘20 [130], Capowski ‘19 [49]. Kallman ‘20 [192]
			Day 6–16	Fligor ‘20 [131]
			Day 6–18	Browne ‘17 [107], Deng ‘18 [169], Guo ‘19 [188], Hallam ‘18 [145], Kobayashi ‘18 [129], Kuwahara ‘15 [127], Li ‘21 [205], Liu ‘20 [208], Liu ‘21 [207], Phillips ‘18 [224], Wang ‘21 [246], Zeng ‘21 [252], Zou ‘19 [259]
		2.2 nM	Day 6–18	Peskova ‘20 [223]
		2.25 nM	Day 6–18	Chichagova ‘20 [48], Georgiou ‘20 [146]
CHIR99021	Wnt signaling activator through inhibition of GSK-3β	3 µM	Day 15–18	Aparicio ‘17 [74], Arno ‘16 [161], Browne ‘17 [107], Lane ‘20 [202], Nakano ‘12 [154], Pan ‘20 [113], Parfitt ‘16 [221], Zheng ‘20 [257]
			Day 15–21	Döpper ‘20 [108]
			Day 18–24 *	Guo ‘19 [188], Hallam ‘18 [145]
		4 µM	Day 18–24	Georgiou ‘20 [146]
		10 µM	Unknown	Luo ‘18 [214], Luo ‘19 [213]
		3 mM	Day 14–17	Wiley ‘16 [248]
			Day 15–18	Sharma ‘17 [230]
			Day 18–21	Kobayashi ‘18 [129]
COCO	A BMP and Wnt inhibitor, and dual modulator of TGF-β signaling	30 µM	Day 0–12	Pan ‘20 [113]
			Day 0–30	Pan ‘20 [113]
DAPT	Inhibitor of notch signaling through inhibition of γ-secretase	10 µM	Day 28–35	Khabou ‘18 [195]
			Day 28–42 *	Eldred ‘18 [177], Lu ‘20 [211], Zerti ‘20 [253]
			Day 29–42	Shrestha ‘19 [232]
			Day 29–45	Wahlin ‘17 [245]
			Day 42–49	Garita-Hernandez ‘20 [186], Garita-Hernandez ‘21 [184]
			Day 44–50	Garita-Hernandez ‘18 [185]
			Day 60–72	Zerti ‘20 [253]
			Day 90–102	Zerti ‘20 [253]
		10 mM	Day 30–40	Sharma ‘17 [230], Wiley ‘16 [248]
DKK-1	Inhibitor of Wnt signaling through binding of Fz	10 ng/mL	Day 0–7	Zhu ‘18 [109]
			Day 18–21	Singh ‘15 [234]
		20 ng/mL	Day 28–35	Singh ‘19 [235], Singh ‘21 [236]
		100 ng/mL	Day 2–4	Meyer ‘11 [135]
			Unknown	Luo ‘18 [214], Luo ‘19 [213]
Dorsomorphin	BMP inhibitor targeting ALK-2, -3, and -6 inhibition	100 ng/mL	Day 2–4	Meyer ‘11 [135]
EC23	Synthetic retinoid	0.3 µM	Day 18–41	Völkner ‘16 [101]
			Day 25–120	Völkner ‘21 [244]
FGF2	FGF signaling ligand	10 ng/mL	Day 14–21	Khabou ‘18 [195], Reichman ‘14 [143]
			Day 21–28	Reichman ‘14 [143]
			Day 28–35	Diakatou ‘21 [171], Freude ‘20 [180], Gagliardi ‘18 [181], Garita-Hernandez ‘18 [185], Garita-Hernandez ‘20 [186], Garita-Hernandez ‘21 [184], Rabesandratana ‘20 [158], Reichman ‘14 [143], Reichman ‘17 [156], Scholler ‘20 [229], Slembrouck-Brec ‘18 [157], Slembrouck-Brec ‘19 [150]
FGF9	FGF signaling ligand	10 ng/mL	Day 21 onwards	Singh ‘15 [234]
			Day 35 onwards	Singh ‘19 [235], Singh ‘21 [236]
IGF1	Interacts with insulin receptors to regulate downstream signaling pathways such as Akt and ERK	5 ng/mL	Day 0–35	Chichagova ‘19 [162], Chichagova ‘20 [48]
			Day 0–37 *	Collin ‘19 [163], Collin ‘19 [164], Dorgau ‘18 [173], Dorgau ‘19 [172], Felemban ‘18 [178], Mellough ‘15 [148], Mellough ‘19 [217], Zhang ‘20 [255], Zhang ‘21 [256]
			Day 2–29	Zerti ‘20 [253]
		10 ng/mL	Day 0–7	Zhu ‘18 [109]
			Day 18–21	Singh ‘15 [234]
			Day 30 onwards	Zerti ‘20 [253]
			Day 35 onwards	Chichagova ‘19 [162], Chichagova ‘20 [48]
			Day 37–90	Collin ‘19 [163], Collin ‘19 [164], Dorgau ‘18 [173], Dorgau ‘19 [172], Felemban ‘18 [178], Mellough ‘15 [148], Mellough ‘19 [217], Zhang ‘20 [255], Zhang ‘21 [256]
			Day 42–200	Kaya ‘19 [193], Kelley ‘20 [194], Kruczek ‘21 [198]
		20 ng/mL	Day 20–30	Regent ‘20 [227]
			Day 21/28 onwards	Kaya ‘19 [193], Kelley ‘20 [194], Kruczek ‘21 [198]
			Day 28–35	Singh ‘19 [235], Singh ‘21 [236]
			Day 35 onwards	Regent ‘20 [227]
IWR1e	Inhibitor of Wnt signaling through stabilization of AXIN	3 nM	Day 0–12	Sharma ‘17 [230], Wiley ‘16 [248]
		2 µM	Day 0–7	Zhu ‘18 [109]
		3 µM	Day 0–6	Döpper ‘20 [108]
			Day 1–6	Eldred ‘18 [177], Wahlin ‘17 [245]
			Day 1–8	Lu ‘20 [211]
			Day 0–12 *	Arno ‘16 [161], Eastlake ‘19 [176], Gao ‘20 [183], Kaewkhaw ‘15 [191], Lane ‘20 [202], Nakano ‘12 [154], Pan ‘20 [113], Parfitt ‘16 [221], Völkner ‘16 [101], Zheng ‘20 [257]
			Day 0–18	Browne ‘17 [107]
			Day 2–12	Aparicio ‘17 [74], Browne ‘17 [107]
			Day 2–14	Dulla ‘18 [174]
		3 mM	Day 2–20	Khan ‘20 [196]
LDN193189	Inhibitor of BMP signaling through inhibition of ALK-2, -3, and -6 receptors	100 nM	Day 0–6	Döpper ‘20 [108]
			Day 0–7*	Zhu ‘18 [109]
		3 µM	Day 0–6	Browne ‘17 [107]
Noggin	TGF-β signaling ligand	10 ng/mL	Day 0–7	Zhu ‘18 [109]
		100 ng/mL	Day 0 onwards	Singh ‘15 [234], Singh ‘19 [235], Singh ‘21 [236]
			Day 2–4	Meyer ‘11 [135]
Rock Inhibitor (Y-27632)	Inhibitor of Rock	10 µM	Day 0–1*	Achberger ‘19 [159], Aparicio ‘17 [74], Browne ‘17 [107], Cora ‘19 [165], Cowan ‘20 [152], Lai ‘21 [199], Lam ‘20 [201], Shrestha ‘19 [232], Zerti ‘20 [253], Zerti ‘21 [254]
			Day 0–2	Collin ‘19 [163], Collin ‘19 [164], Dorgau ‘18 [173], Dorgau ‘19 [172], Felemban ‘18 [178], Georgiou ‘20 [146], Hallam ‘18 [145]
			Day 0–14	Dulla ‘18 [174]
			Day 24–25	Achberger ‘19 [159], Cora ‘19 [165]
		20 µM	Day 0–1	Döpper ‘20 [108], Kaya ‘19 [193], Kelley ‘20 [194], Kruczek ‘21 [198], Regent ‘20 [227], Zheng ‘20 [257]
			Day 0–4	Kaewkhaw ‘15 [191]
			Day 0–6	Deng ‘18 [169], Guo ‘19 [188], Kobayashi ‘18 [129], Kuwahara ‘15 [127], Wang ‘21 [246], Zeng ‘21 [252], Zou ‘19 [259]
			Day 0–12	Eastlake ‘19 [176], Gao ‘20 [183], Lane ‘20 [202], Nakano ‘12 [154], Pan ‘20 [113], Völkner ‘16 [101]
			Day 0–18	Li ‘21 [205], Liu ‘20 [208], Liu ‘21 [207], Parfitt ‘16 [221]
			Day 0–20	Khan ‘20 [196]
		10 mM	Day 0–1	Shimada ‘17 [231]
		20 mM	Day 0–12	Sharma ‘17 [230], Wiley ‘16 [248]
SAG	Hedgehog pathway activator through activation of Smo	100 nM	Day 8–16	Eldred ‘18 [177]
			Day 10–18	Lu ‘20 [211]
			Day 12–18	Eastlake ‘19 [176], Gao ‘20 [183], Kaewkhaw ‘15 [191], Völkner ‘16 [101], Wahlin ‘17 [245]
			Day 12–20	Khan ‘20 [196]
			Day 14–17	Wiley ‘16 [248]
			Day 14–20	Dulla ‘18 [174]
			Day 15–18	Aparicio ‘17 [74], Arno ‘16 [161], Browne ‘17 [107], Lane ‘20 [202], Nakano ‘12 [154], Pan ‘20 [113], Parfitt ‘16 [221], Sharma ‘17 [230], Zheng ‘20 [257]
SB431542	Inhibitor of TGF-β signaling through inhibition of ALK-4, -5, and -7 receptors	3 µM	Day 0–6	Browne ‘17 [107]
		10 µM	Day 0–6	Döpper ‘20 [108]
			Day 0–7*	Zhu ‘18 [109]
SU5402	Inhibitor of FGF signaling through inhibition of fibroblast growth fact receptor 1 (FGFR-1)	2.5 µM	Day 18–24	Chichagova ‘20 [48], Georgiou ‘20 [146]
		5 µM	Day 15–21	Döpper ‘20 [108]
			Day 18–24*	Guo ‘19 [188], Hallam ‘18 [145]
		10 µM	Day 0–10	Meyer ‘09 [144]
			Day 16–40	Meyer ‘09 [144]
		3 mM	Day 18–21	Kobayashi ‘18 [129]
Wnt3a	Wnt signaling activator	100 ng/mL	Day 0–10	Meyer ‘09 [144]
XAV939	Wnt signaling inhibitor through stabilizing AXIN	100 ng/mL	Day 2–4	Meyer ‘11 [135]

### 4.2. Comparison of Specific Signaling Pathway Modulators and Their Effect on Retinal Organoid Differentiation

**Figure 9:**
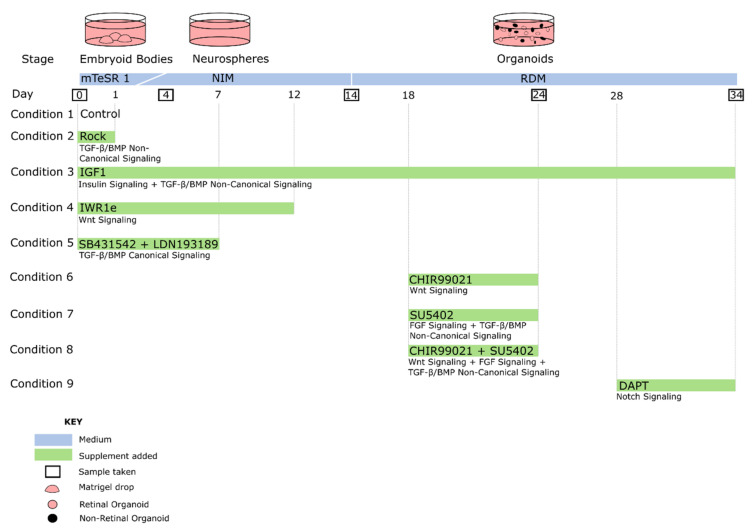
Experimental outline of pilot. A collection of frequently used agonists and antagonists for different signaling pathways reviewed in this article were systematically tested for their efficiency at directing retinal development. Adapting our previously published protocol [149], stem cell clumps were embedded in 3D Matrigel drops for 4 days, transitioning from mTeSR1 medium to neural induction medium (NIM). At day 4, they were removed from the gel and plated in a 2D environment for a further 10 days to allow the embryoid bodies to enter the anterior neural fate. Once neurospheres had formed, they were scraped off and cultured 3D in a floating environment in retinal differentiation medium (RDM) to form organoids. Condition 1 was the control condition, with no supplements added. Condition 2 consisted of treatment with Rock Inhibitor from day 0 to 1 only. Condition 3 included a continuous treatment of IGF1 from day 0 to 34. Condition 4 consisted of IWR1e treatment from day 0 to 12. Condition 5 included a double treatment of SB431542 and LDN193189 from day 0 to 7. Conditions 6–8 all included treatments from day 18 to 24 consisting of CHIR99021, SU5402, and CHIR99021 + SU5402, respectively. Finally, condition 9 consisted of treatment with DAPT from day 28 to 34. All culture conditions were kept until day 34, and samples were taken at day 0, 4, 14, 24 and 34 for analysis. (Pilot study: *n* = 1 for all conditions).

After collating the results of the literature search, we selected a range of conditions that were representative of the methods we examined and performed a pilot experiment using our previously published protocol [149] (outlined in Figure 9). We chose conditions that spanned retinal development, from initial time points when embryoid bodies and neurospheres form (day 0–14), to later stages when 3D organoids start to form and mature (day 14 onwards). We observed retinal organoid development by brightfield microscopy, and took samples at day 0, 4, 14, 24 and 34 to measure the expression of key retinal developmental genes such as *PAX6*, *RAX* and *VSX2*.

#### 4.2.1. Regulation of Signaling Pathways by External Factors Affecting Retinal Development

To make an inventory of the effect that (ant-) agonists have on the key signaling pathways (Figure 2, Figure 3, Figure 4, Figure 5 and Figure 6) in retinal organoid development, we tested a range of conditions spanning the early and late regulation of these pathways in a proof-of-concept experiment (Figure 9). In separate cultures, we added supplements affecting early retinal development (between days 0 and 12) (Figure 9), including Rock Inhibitor, IGF1, IWR1e and the combination of SB431542/LDN193189. Similarly, we treated cultures with supplements affecting the later stages of development (between days 18 and 34, Figure 9), by adding CHIR99021, SU5402, the combination CHIR99021/SU5402 and, finally, DAPT. All conditions were systematically and simultaneously evaluated by microscopy and RT-PCR, as described below.

**Figure 10:**
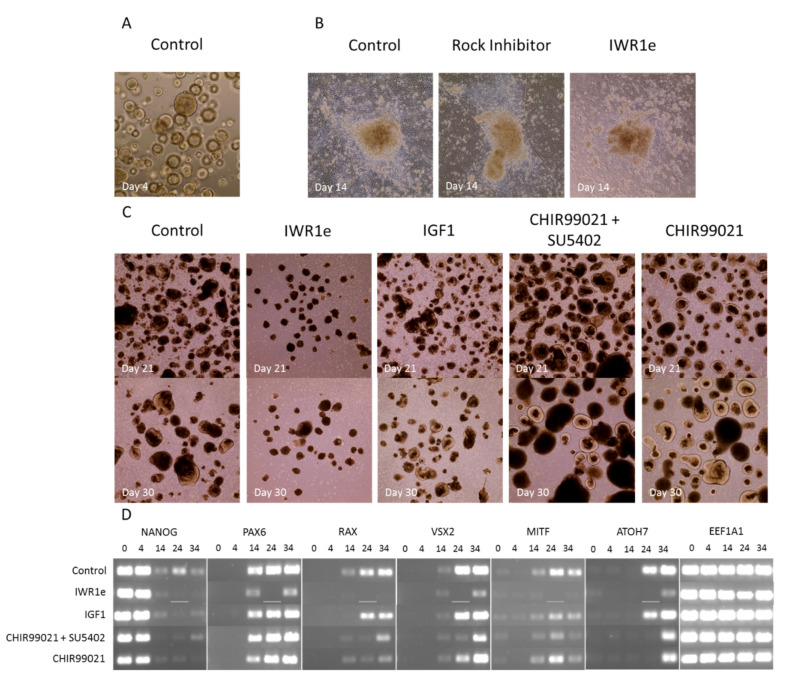
Changes in retinal development through microscopy and gene expression assay. (**A**) All conditions developed comparable embryoid bodies to the control up to day 4. (**B**) Throughout the next 2D stage of generating neurospheres (day 4–14), changes between conditions were observed. Rock Inhibitor-treated cells had two distinct types of epithelial outgrowth, with a border separating them, whereas IWR1e-treated cells resulted in poor epithelial outgrowth and an increase in cell death. (**C**) Only IWR1e, IGF1, CHIR99021 + SU5402, and CHIR99021 treatments showed significant changes in retinal organoid yield compared to the control. IWR1e treatment severely impact retinal organoid yield and the overall organoid size. Continuous IGF1 treatment resulted in almost double the number of retinal organoids compared to the control. CHIR99021/SU5402 double treatment increased retinal organoid yield 2.5-fold; however, individual CHIR99021 treatment gave the best yield with a 5-fold increase. (**D**) The differences between conditions were also largely reflected in the gene expression of key developmental markers such as *PAX6*, *RAX* and *VSX2*, as well as cell-specific markers such as *MITF* and *ATOH7*. Differences of retinal development genes were observed between conditions over time (Day 0, 4, 14, 24, 34). Unfortunately, IWR1e treatment resulted in poor organoid yields and subsequently, there was less RNA available for day 24, as represented by the *EEF1A* reference gene sample.

In the embryoid body stage from day 0 to 4, we did not observe significant developmental differences between culture additions and the control. All conditions (control, Rock Inhibitor, IGF1, IWR1e, SB431542/LDN193189, CHIR99021, SU5402, CHIR99021/SU5402, DAPT) produced healthy EBs (Figure 10A). It is important to note that in CHIR99021, SU5402, CHIR99021/SU5402 and DAPT conditions, the EB stage (day 0–4) is the same as the control conditions, due to these additions being added at later time points, as depicted in Figure 9. In contrast, during the second stage of development, from day 4 to 14 when the EBs are plated in a 2D environment (Figure 9), a number of changes were observed in the IWR1e, Rock Inhibitor, and SB431542 + LDN193189-treated cells compared to the control (Figure 10B). The addition of IWR1e inhibited the outgrowth of the neurospheres, with the epithelial outgrowth barely leaving the neural center of the neurosphere after 10 days. There was also increased cell death in this condition, defined by brightfield microscopy, when compared to the control, also seen in SB431542 + LDN193189-treated cells. Rock Inhibitor-treated cells displayed two types of epithelial outgrowth, with distinct borders forming between the two, which no other condition exhibited. All the remaining conditions (IGF1, CHIR99021, SU5402, CHIR99021 + SU5402, and DAPT) developed comparably to the control during the neurosphere stage (Supplementary Figure S1).

Organoids generally develop into a retinal or non-retinal fate between days 20 and 25, at which point they should be separated from each other. In our test, the yield of retinal organoids was measured with brightfield microscopy during this final stage at day 25 (Table 2), and varied greatly between various culture conditions tested. We found that some conditions behaved similarly to the control: the treatment of SU5402 or DAPT had no apparent effect on retinal organoid yield (Table 2), whilst some supplements added during the initial phase of development, from day 0 to 12, harmed the yield of retinal organoids. IWR1e-treated cells did not form retinal organoids, and only formed around half the number of total (retinal and non-retinal) organoids compared to the control (Table 2). The surviving organoids were also significantly smaller, most likely the result of the increased cell death observed during the 2D stage (Figure 10C). Rock Inhibitor and SB431542/LDN193189-treated cultures had similar numbers of total organoids compared to the control, but with fewer retinal organoids, resulting in a lower yield. IGF1 was added throughout the culture time, and had a positive effect on retinal development, with almost double the yield of retinal organoids by day 25. The treatment with CHIR99021 boosted retinal organoid yield five-fold, whilst also generating more than double the total amount of organoids compared to the control (Table 2). Organoids made by CHIR99021 treatment showed characteristic golden laminated structures that were present throughout longer-term culturing. Initial data from our laboratory suggest long-term retinal development is not affected by CHIR99021 treatment (Wagstaff et al., unpublished). Adding SU5402 in combination with CHIR99021 only negatively added to the outcome, with a retinal yield only 2.5-fold higher than the control, whilst adding SU5402 alone had no apparent effect compared to the control (Table 2, Figure S1).

#### 4.2.2. Variable Retinal Development Confirmed by Gene Expression Changes of Key Markers

We took organoid samples at pivotal times throughout development: day 0, day 4, day 14, day 24, and day 34. These coincided with the end of the embryoid body stage (day 4), the end of the neurosphere stage (day 14), and the period when retinal organoids start to develop separately from the non-retinal organoids (day 24). We analyzed the RNA from the samples by RT-PCR for the presence and changes in gene expression of crucial retinal genes (Figure 10D). In the developing retinal organoid control [149], the stem cell marker *NANOG* was highly expressed on days 0 and 4, before decreasing on days 14, 24, and 34. The early retinal markers *PAX6* and *RAX* and the optic cup marker *VSX2* were all present from day 14 onwards, with *VSX2* expression increasing between day 14 and 24. The RPE-specific marker *MITF* was expressed throughout development, peaking at day 24. Finally, the retinal ganglion cell marker *ATOH7* was highly expressed from day 24 onwards. In line with the results of retinal organoid yields, treatment with SU5402 or DAPT did not appear to change the expression of key retinal developmental genes compared to the control. Interestingly, all conditions resulted in lower levels of the stem cell marker *NANOG* when compared to the control in days 14 to 34, suggesting an improved transition from a stem cell state to differentiation into the neural lineage.

As described above, the addition of IWR1e produced the lowest retinal organoid yield (Table 2), and this is reflected in its gene expression (Figure 10D). IWR1e treatment leads to a complete loss of *RAX* throughout the culture, and a decrease in expression of the retinal lineage markers *PAX6* and *VSX2*. The RPE-specific marker *MITF* expression was present, and the ganglion-specific *ATOH7* was severely reduced, with a lowered expression at day 24 and a large decrease at day 34. Treatment with Rock Inhibitor or SB431542/LDN193189 had no apparent effect on retinal gene expression, even though retinal organoid yield was decreased. IGF1 treatment resulted in a delay of *RAX* expression, with *RAX* only present from day 24 onwards. However, the expression of other markers, upon prolonged IGF1 treatment, remained unchanged throughout development compared to the control. Prolonged IGF1 treatment appeared to slightly increase the yield of retinal organoids (Table 2). The condition with the best retinal yield, CHIR99021 treatment, unexpectedly exhibited a slight decrease in *RAX* expression, although *PAX6* and *VSX2* expression was comparable to the control. Furthermore, there was a delay in the expression of *ATOH7*, with *ATOH7* being absent until day 34. This was mirrored in CHIR99021 and SU5402 double treatment, where *ATOH7* was not expressed until day 34, alongside an apparent decrease in *VSX2* expression (Figure 10D).

### 4.3. Comparison of Cell Type-Specific Expression during Retinal Organoid Development

As presented above, the many different methods used to generate retinal organoids gives rise to increased variability between cultures, making methods hard to compare. Techniques such as RNA-seq allow us to quantify the amount of cell type-specific RNA present in cultures, which gives us an idea of cell types present in the diverse organoid stages, and the gene expression similarities and differences between protocols. We can also estimate how representative these datasets are for human retinal development. Datasets have been generated from multiple stem cell lines used to make retinal organoids and fetal retinal cultures [8,191,197]. We analyzed these three datasets for the expression of relevant cell type-specific markers, and aligned them both to the RT-PCR data presented in this manuscript, and the RNA-seq data from our control retinal organoid differentiation protocol [149]. At this point, it is important to note that due to the different methods and analysis used, these data cannot be directly compared, but can act only as an indication of the cell types present and when they appear. For reason of comparison (cells in their full physiological context), we focused on expression studies of whole retinal organoids.

**Figure 11:**
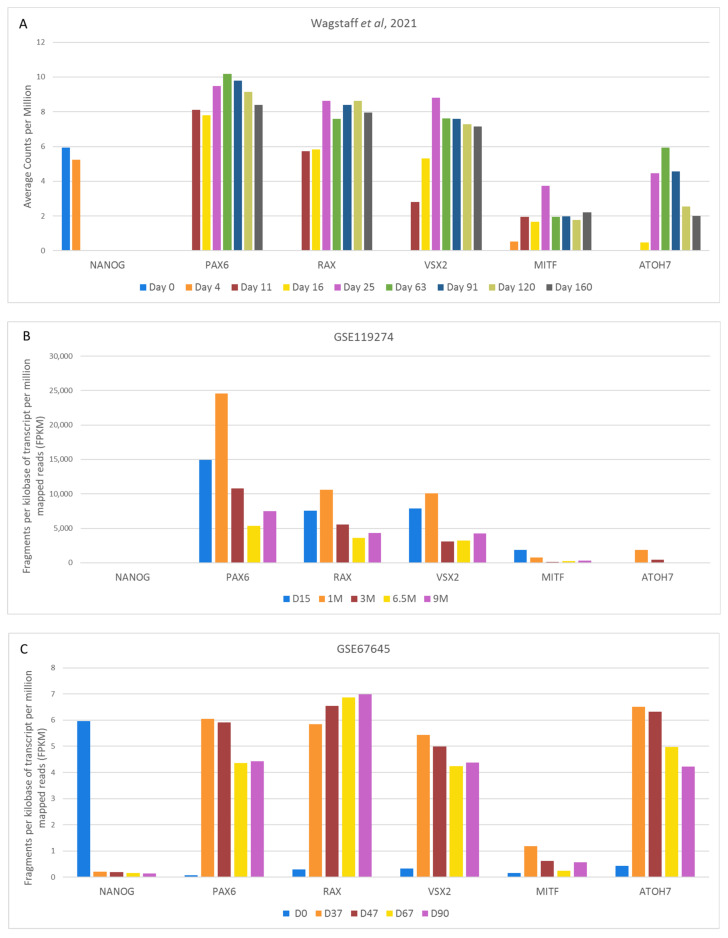

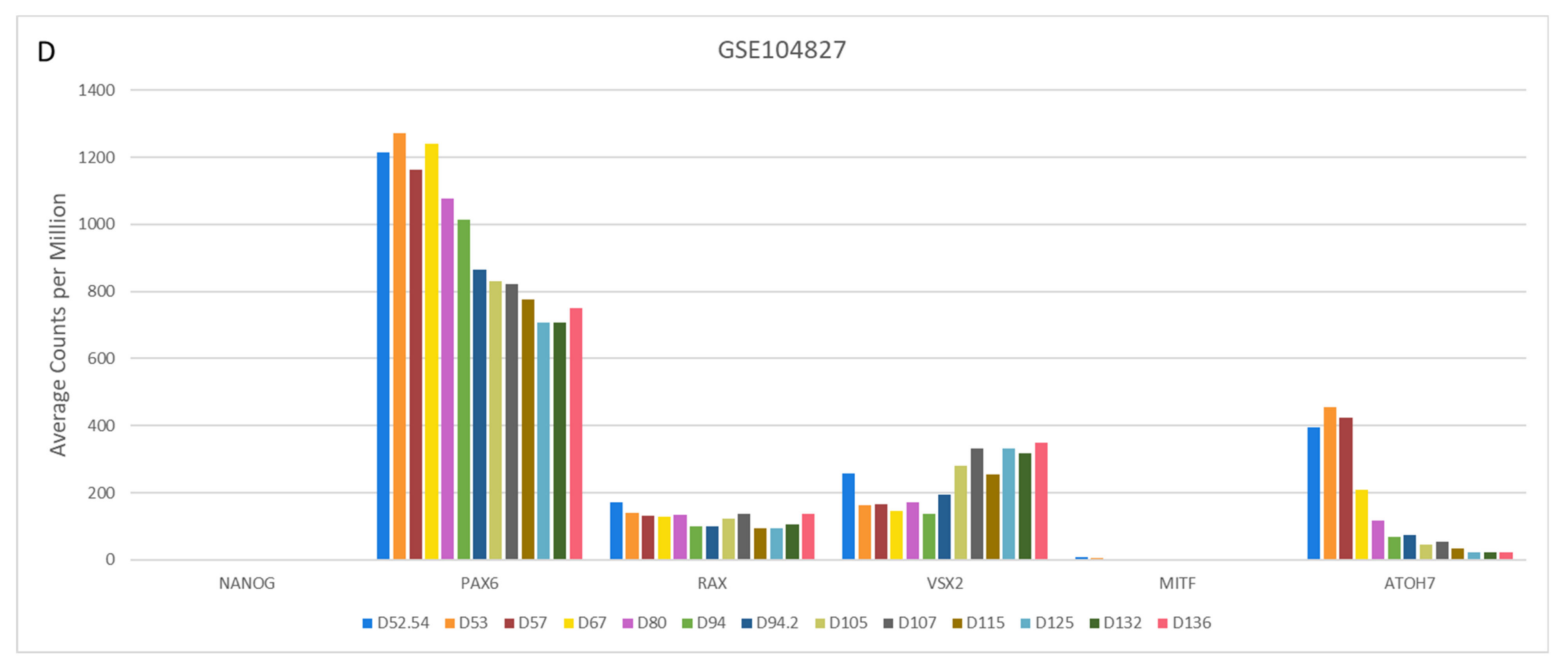
RNA-seq analysis of differing methods of in vitro retinal development, in addition to in vivo fetal retinal development. We compared the gene expression of our pilot experiment to RNA-seq analysis of different retinal organoid differentiations and fetal retina samples. (**A**) Our previously published protocol using H1 embryonic stem cells shared similar gene expression with our pilot experiment shown in this manuscript over the course of the first 34 days. (**B**) A different method of generating retinal organoids using the H1 embryonic stem cell line also showed expression of key retinal genes increasing over the first month, comparably to our pilot experiment and RNA-seq. (**C**) Retinal organoids generated from a different embryonic stem cell line (H9) showed variable expression levels of key genes when compared to A and B. Some genes, such as *RAX*, increased over time in line with other RNA-seqs; however, other genes, such as *VSX2*, unexpectedly decreased over time. (**D**) RNA-seq of fetal retina presented surprising results. After being expressed by day 10, *PAX6* and *RAX* both decreased until day 69, before significantly increasing along with *VSX2*. This was not shown in RNA-seqs of retinal organoids, where *PAX6* and *RAX* expression slowly increased throughout earlier time points, not decreased.

We initially compared the RT-PCR data from this manuscript with RNA-seq data we previously published generating retinal organoids from H1 embryonic stem cells. In general, we found that the appearance of retinal genes in the control condition in our pilot experiment described in this manuscript matched the control retinal organoid differentiation RNA-seq data we previously published (Figure 11A): *NANOG* was present on days 0 and 4 before losing its expression. *PAX6* was the first neural developmental gene to be expressed, followed by the retinal marker *RAX*, with *VSX2* being expressed last. *MITF* expression seemed to peak at day 25 before decreasing, as seen in our pilot control condition. *ATOH7* was expressed from day 25 onwards. Dataset GSE119274 [197], presented in Figure 11B, originated from a study that also used the embryonic stem cell line H1 to generate retinal organoids, taking samples from day 15, 1 month, 3 months, 6.5 months and 9 months in culture. We observed that *PAX6* is also the first retinal developmental marker to appear in this method, peaking after 1 month of culture, with *RAX* and *VSX2* being similarly expressed at this time point, similarly to our RT-PCR and RNA-seq data. We also observed a significant increase in the expression of *ATOH7* between 15 days and 1 month of culture, mirroring our RT-PCR analysis of *ATOH7* in our cultures. Another group, Kaewkhaw et al. (2015), cultured organoids and generated the dataset GSE67645 [191]. Their RNA-seq for the relevant data is presented in Figure 11C. These authors generated retinal organoids using another embryonic stem cell line, H9, and analyzed samples taken throughout the culture at days 0, 37, 47, 67 and 90. They showed higher levels of *PAX6*, *RAX* and *VSX2* expression at day 37 compared to the cell-specific marker *MITF*, something we also observed in our cultures. Similarly, the expression of *ATOH7* increased significantly between day 0 and 37, in line with our findings. Finally, we analyzed the transcriptomic dataset GSE104827 [8] of the developing fetal human retina (presented here in Figure 11D). Extensive data from samples were available throughout development (day 52 or 54, 53, 57, 67, 80, 94, 94 (second sample), 105, 107, 115, 125, 132, 136). Ideally, in vitro retinal development should be as close to fetal expression as possible. However, it is often difficult to compare these due to the hugely different environments and timelines. The most striking difference was the large increase in total expression when compared to the data of our retinal organoids (Figure 11A), with some genes expressed 100-fold more. Although it was difficult to compare all of the different organoid datasets with the fetal retina, we did observe some interesting similarities. In dataset GSE104827, *PAX6* expression was at its highest after around 50 days of development, before decreasing over time to day 136. In our retinal organoid RNAseq data, we also observed a peak in *PAX6* expression after around 60 days of development, before decreasing over the course of the differentiation to day 160. The fetal expression of *RAX* slightly increased and decreased throughout development, but remained somewhat constant between day 52 and 136. This was mirrored in our organoid development, varying slightly but keeping relatively stable over time. Interestingly, we observed that *VSX2* expression in fetal retina increased over time from day 53 to 136, whereas in all three organoid datasets, *VSX2* expression was at its peak within the first month of development, before decreasing over time during the culture. Finally, we found that although *ATOH7* expression in the fetal retina did mirror the expression found in our dataset, the peak of expression was shifted to an earlier time point. In our RNAseq dataset, *ATOH7* expression increased gradually until day 63, where it peaked before decreasing over time. However, in the fetal retina, *ATOH7* expression peaked by day 53, and by day 67 was already reduced by around half, decreasing further throughout development. When comparing these similarities in gene expression, it is important to consider the relative timeline, as retinal organoid maturation and fetal eye maturation do not occur within the same timeframe. Taken together, these data highlight important similarities and differences between in vitro and in vivo retinal development.

## 5. Discussion and Conclusions

Since the introduction of retinal organoid technology, there has been a need to emulate in vivo retinal development as closely as possible in vitro. To achieve this, research has been increasingly focused on the temporal identification of mapping the molecular changes that cells undergo during retinal development. This has culminated in numerous protocols that vary greatly in their method and as such, are difficult to compare. This review systematically, and for the first time, explores the many different methods that are used to generate retinal organoids. It includes an inventory and comparison of the origin, signaling pathways and (ant-) agonists used in the literature. Furthermore, we systematically tested a range of additions reviewed here using our own protocol [149] to corroborate the possible effect they had on organoid development.

Our review shows that a significant proportion of current methods originate from three main protocols [127,154,155]. Using these as a base, researchers have continuously improved methods to influence in vitro development by regulating important signaling pathways present throughout in vivo embryonic development. These pathways include the TGF-β/BMP, hedgehog, Wnt and notch signaling pathways. By manipulating these signaling pathways, using (ant-)agonists (IWR1e, BMP4, CHIR99021), researchers aim to establish a more reliable and reproducible way of generating organoids that perfectly mimic the in vivo situation. We systematically looked at a number of conditions that further contribute to the improvement and consistency of the existing protocols. In experimental conditions, we observed that a lot of the most commonly used additions, such as IWR1e, SB431542/LDN193189, and SU5402, did not positively affect retinal development in our protocol except for CHIR99021 treatment, which significantly improved retinal organoid yield. However, it is important to note here that our pilot experiments reported here, do not conclude that the commonly used additions such as IWR1e have no effect in other methods of generating retinal organoids and/or animal models of retinal development. It is also important to note that we present preliminary findings in a pilot experiment about the effects these different signaling molecules have on retinal organoid development. For more definitive conclusions, extensive further studies are needed. The large variation between protocol methods, coupled with the increasing variability between different cell lines means that further experiments should be carried out to optimize culture conditions.

### 5.1. The Use of Different Protocols Gives Rise to Variable and Difficult to Compare Cultures

The majority of the established methods of generating retinal organoids arise from three core protocols, with different (ant-)agonists being used. Embryoid bodies are often created in vastly different environments, including SFEBq [154], free floating EB formation [155], and a 3D Matrigel suspension [149]. In SFEBq cultures, stem cells are dissociated to single cells and quickly re-aggregated in low adhesion 96-well plates with a defined number of cells per well. In free floating EB formation, stem cells are dissociated into small clumps and cultured in suspension, where they aggregate to form EBs. Three-dimensional Matrigel suspension also dissociates stem cells into small clumps, before embedding them in Matrigel drops (Figure 9), where they form EBs. These conditions are also continuously being optimized and innovated, from controlling embryoid body size by counting the number of starting cells, to generating microwell systems that can result in hundreds of similar EBs [152]. This variation in method can affect the efficiency of external factors on your culture, as we observed.

IWR1e and IGF1 are two of the most commonly used external factors added to retinal organoids cultures (Table 1). However, it is important to note that some supplements that are regularly added may come from a single well-replicated protocol and might not be as effective throughout all methodologies. For instance, over two-thirds of the times IWR1e is used, it is added between day 0 and 12 at a concentration of 3 µM, showing a consistently positive effect on retinal development. This concentration and timeline come from Nakano et al. (2012), one of the most popular methods to use by multiple different groups. Therefore, although the use of IWR1e is widespread and popular, it can be traced back to a specific protocol. In our protocol, however, using IWR1e between day 0 and 12 at 3 µM resulted in a complete loss of retinal identity based on brightfield microscopy and RT-PCR, as well as fewer organoids overall. IGF1, on the other hand, has been added at multiple time points throughout organoid development at various concentrations, depending on the protocol used. In our hands, it had a positive effect, increasing the yield of retinal organoids. The results we observed for the IWR1e treatment are drastically different to other methods, which could, in turn, be explained by our method of choice. To generate our embryoid bodies, we use a 3D Matrigel encasement method, described previously [149]. Matrigel is well known for being a semi-defined substrate full of extracellular proteins and factors. The presence of these proteins in the initial culture could not only affect native cell–cell interactions and signaling in the control conditions, but also could exert a conflicting effect on the initial embryonic signaling to some external factors. These drastic differences show how, just as retinal yields can be cell-line-dependent, small molecule effects are not only time- and concentration-dependent, but also method-dependent, and should be adapted to individual protocols.

### 5.2. Healthy Early Development of Embryoid Bodies Is Not a Guarantee for Healthy Organoids

One of the most important steps of generating proper retinal organoids as a reliable model, with long-term lamination and all retinal cell types present and matured, is to have good starting material. Organoids generated from imperfect stem cells or embryoid bodies tend to lose their retinal identity throughout development. They usually also affect the surrounding organoids and thus, the entire culture. The importance of initial EB development has been previously shown by systematically testing mechanical, enzymatic and dissociation–reaggregation methods of generating embryoid bodies from stem cells in parallel [148]. Using these methods, the EBs were cultured either in the presence or absence of Rock Inhibitor for the first 48 h. Although Rock Inhibitor aided embryoid body formation, there was no impact on early retinal specific gene expression. Interestingly, we also observed no significant changes in retinal yield or gene expression in the Rock Inhibitor-treated and control retinal organoid cultures.

In line with this, we did not observe any negative effects during the initial stage of embryoid body formation (day 0 to 4) for any of our conditions tested, with all cultures comparable to the control (Figure 10). In contrast, during the second stage of development characterized by the formation of neurospheres (day 4 to 14), we observed reduced neuroepithelial outgrowth in cultures treated by IWR1e, and increased cell death in both IWR1e-treated and SB431542/LDN193189 double-treated cells. These both resulted in cultures with very few retinal organoids, with a significantly reduced size.

### 5.3. One Pathway, One Agonist/Antagonist?

The analysis and comparison of different protocols showed that there are most likely multiple supplements that affected the same pathway. Are all of these different factors needed, or are some redundant? This, again, depends on the model that is being used. As we have shown, the inhibition of the Wnt pathway at an early stage in development by either DKK1 or IWR1e is commonly used to increase retinal organoid yield (Table 1). Both supplements are usually added within the first two weeks of development but inhibit Wnt signaling through different substrates of the Wnt signaling pathway. DKK1 affects the upstream binding of *Frizzled* to LRP5/6, preventing the formation of the *Frizzled*–LRP5/6 complex on the cell surface; IWR1e inhibition occurs downstream by stabilizing Axin, part of the destruction complex within the cell (Figure 5). Interestingly, mutations in Drosophila *Frizzled* cause defects in the polarity of the fly eye [266]. In murine models, *Frizzled* knockouts cause increased cell death, incomplete closure of the ventral fissure, and late-onset progressive retinal degeneration [267]. Therefore, when exploring the effect of the upstream *Frizzled* mutations in retinal organoid development, the possible upstream DKK1-mediated effect should be taken into account.

In addition to the aforementioned multiple Wnt inhibitors, we also observed that multiple activators of the Wnt pathway (Figure 5) have been used in retinal organoid protocols. For example, Wnt3a and CHIR99021 (Table 1) both activate Wnt signaling. However, similarly to DKK1 and IWR1e, they effect different parts of the pathway. Wnt3a acts upstream, and is added as a recombinant protein, acting as a ligand that binds to *Frizzled* and activates Wnt signaling. In contrast, CHIR99021 acts downstream, and is a GSK-3β inhibitor that activates Wnt signaling from inside the cell.

A number of other supplements affect one single pathway in slightly different ways. For example, in the BMP pathway (Figure 3) the antagonist noggin acts slightly more upstream than dorsomorphin: noggin binds to specific ligands in a competitive manner, preventing them from binding to cell surface receptors. Dorsomorphin, on the other hand, inhibits the receptors themselves. The action can be very specific and subtle. For example, dorsomorphin inhibits ALK-2, -3, and -6 receptors, whereas its derivative, LDN193189, only inhibits ALK-2 and -3 receptors. However, to complicate matters, LDN193189 is significantly more potent than dorsomorphin, allowing researchers to gain a similar affect with smaller concentrations of LDN193189 than dorsomorphin. A final example of signaling complexity is the IGF1 treatment: IGF1 affects the MAP kinase and PI3 kinase pathways, which are a part of the non-canonical branch of the TGF-β/BMP signaling pathway (Figure 3). However, IGF1 does not act through the traditional TGF-β/BMP receptors, as it binds to the insulin-like growth factor 1 receptor (IGF1R), allowing for an alternative way of regulating the TGF-β/BMP signaling pathway.

### 5.4. Does the Synergistic Addition of Multiple Factors Result in an Additive Effect, or Is It Not Necessary?

We noticed in the literature that a subset of factors were commonly added together to target a single pathway simultaneously, or influence multiple pathways. For example, dual SMAD inhibition by SB431542 and LDN193189 is used to regulate both the branches of the TGF-β/BMP signaling pathway, and we found that they were added exclusively together in retinal organoid protocols [107,108] (Table 1). The suppression of TGF-β and BMP signaling has also been achieved by SB431542 and dorsomorphin treatment and is used in vitro and in vivo to direct cells into the neural and retinal fates [105,268,269]. Yet, other factors such as CHIR99021 and SU5402, affecting the Wnt and FGF pathways, respectively, were either used together or individually in different differentiation methods. To investigate the potential effect of the dual inhibition of these factors, we experimentally tested CHIR99021 and SU5402 additions individually, as well as together (Figure 9). We found that CHIR99021 treatment positively influenced retinal organoid development in our protocol, whereas SU5402 treatment did not (Table 2, Figure S1). In the dual treatment, the positive effects of CHIR99021 were less pronounced, with fewer retinal organoids developing and a decrease in the expression of the optic cup marker *VSX2* compared to the individual CHIR99021 treatment. This suggests that in our protocol, SU5402 does not increase the development of retinal cell fate and obstructs the effect of CHIR99021 treatment.

It is important to note that in the overwhelming majority of protocols, more than one (ant-) agonist is used. The number of combinations of different supplements is very large and as such, not every condition can be tested. We broke these conditions down into their separate factors and tested a range of the most commonly used methods, but this does not take into account the synergistic effect that multiple treatments could have. For example, we found that Wnt inhibition during early development had an extremely negative effect on retinal gene expression and organoid yield. However, in protocols that use IWR1e, additional treatment with SAG immediately follows this, which activates hedgehog signaling [113,191,240], something that did not happen during our “individual” conditions. Furthermore, we observed that SU5402 has no significant effect on the development of retinal development in our protocol. However, in other protocols, SU5402 treatment is sometimes preceded by the addition of BMP4 at an earlier time point [48,129,188]. This could explain the differences we see using these additions in our and other organoid development protocols.

### 5.5. Research Implications and Future Perspectives

Here, we presented a review, that is, to our knowledge, the first systematic overview on methods used to generate retinal organoids, focusing on the external treatments used to influence the activity of specific pathways that lead to improved retinal organoid development. We aimed to provide an insight into how researchers are continuously improving their methods to best mimic in vivo development through the regulation of these vital signaling pathways. Although we are still lacking major knowledge in the field of retinal development, constantly evolving methods and techniques such as single-cell RNA-seq are allowing us to map the molecular route that cells take more clearly than ever before. Recent publications presenting single-cell RNA-seq analysis of retinal organoids and human retinas allow us to visualize retinal development in great detail [152]. This, in turn, allows us to recreate the in vivo environment more and more precisely. While we show here an in-depth analysis of treatments used in organoid development, we only provide the most important snapshot of the experimental conditions researchers frequently use. Many combinations are used in conjunction with one another, and these should be addressed in the future to reduce variability between protocols and increase comparability. This review presents a solid base, showing the many additions that are used in retinal organoids protocols, what pathway these work through and how that pathway affects retinal development, that can be built upon in future studies.

## 6. Materials and Methods

### 6.1. Protocol Search

We searched the literature in a comprehensive manner to include all protocols used to generate retinal organoids, which was undertaken using the NCBI publication database PubMed. Searches up to and including 8 May 2021 for the terms “Retinal Organoid” and ““Retinal” AND “Organoid”” identified 449 and 103 results, respectively. We removed duplicate literature entries, and we screened and excluded manuscripts based on the following predefined criteria: (1) that the authors should generate their own retinal organoids, and (2) that these organoids should be made from human stem cell tissue (hESC or hiPSC). Further, the papers should be (3) readily available in original articles and (4) published in English. For example, this meant that papers generating organoids from primary retinal tissue, or papers analyzing publicly available organoid transcriptomes, were discarded. No additional criteria were used in order to include as many methods as possible. In order to check whether we did not miss any articles, after the initial screen, we inspected referenced papers from the selected publication set, and possible relevant remaining articles were included in the final analysis. In total, we identified 127 manuscripts, using 131 methods.

### 6.2. Organoid Generation

Organoids were generated as previously described [149], with one minor change. Briefly, H1 ESCs (WiCell, Madison, WI, USA) were dissociated into smaller clumps (around 100 µm in diameter) using 0.5 mM EDTA in PBS, embedded in Matrigel (Corning, Corning, NY, USA) and plated with 3ml of an mTeSR1 and neural induction medium (NIM) mixture (3:1 ratio). NIM consisted of DMEM/F12 (-L-Glutamine) (1:1), N2 supplement, non-essential amino acids, heparin (2 µg/mL), PenStrep and GlutaMAX. The day of embedment was annotated as day 0, with the medium being changed on day 1, (1:1 ratio), day 2 (1:3 ratio) and day 3 (full NIM). After four days of differentiation, large organized embryoid bodies were formed, which were taken out of the Matrigel with cell recovery solution (Corning, Corning, NY, USA). The whole EBs were then plated on 6-well plates coated with hESC-qualified Matrigel (Corning, Corning, NY, USA), and were incubated for a further 10 days, with NIM media changes every other day. On day 11, the neural centers were carefully dislodged and transferred to a 60 mm dish containing retinal differentiation medium (RDM). RDM consisted of DMEM/F12 (-l-Glutamine) (3:1), B27 supplement, non-essential amino acids, PenStrep and GlutaMAX. Retinal organoids were kept in culture up until day 34, changing RDM medium every other day. For experiments containing supplements (*n* = 1), they were added with every medium change unless stated otherwise as follows: Rock Inhibitor (10 µM—SelleckChem, Houston, TX, USA) from day 0 to 1, IGF1 (5 ng/mL—Thermofisher, Waltham, MA, USA) from day 0 onwards, IWR1e (3 µM—Merck Millipore, Burlington, MA, USA) from day 0 to 12, SB431542 (10 µM—Sigma, St. Louis, MO, USA) + LDN193189 (100 nM—SelleckChem, Houston, TX, USA) from day 0 to 7, CHIR99021 (3 µM—Merck Millipore, Burlington, MA, USA) from day 18 to 24, SU5402 (5 µM—StemCell Technologies, Vancouver, BC, Canada) from day 18 to 24, CHIR99021 + SU5402 double treatment from day 18 to 24, and DAPT (10 mM—Sigma, St. Louis, MO, USA), from day 28 onwards. Supplements were reconstituted according to the manufacturer’s instructions. Carriers were tested previously for adverse effects, of which none were found.

### 6.3. RT-PCR and sqPCR

To generate a snapshot overview of gene expression between the different conditions used, the presence and gene expression of markers of retina specific cell types in the whole organoids was measured. Total RNA was extracted from 5 to 6 organoids per sample using the Qiagen RNeasy Mini kit with a DNase step according to the manufacturer’s instructions. cDNA was generated using Superscript III (Invitrogen, Waltham, MA, USA), and sq-PCR was performed with HOT FIREpol DNA Polymerase (Solis Biodyne, Tartu, Estonia). Input RNA of 50 ng was used for all samples other than IWR1e day 24, where less had to be used due to poor sample yield. Gene expression was compared to the reference gene *EEF1A*. sq-PCR primer sequences can be found in Supplementary Table S1.

### 6.4. RNA-Seq Analysis

To compare relative expression of key marker genes of retinal development in our studies with other (published) datasets, we acquired published RNA-seq datasets from GEO (https://www.ncbi.nlm.nih.gov/geo). In-depth descriptions of different RNA-seq methods can be found in each publication [8,149,191,197].

## Figures and Tables

**Table 2:** Retinal organoid yields. Cultures were analyzed on day 25, and both retinal and non-retinal organoids were manually counted for each condition through brightfield microscopy images. The yield of retinal organoids compared to the total amount of organoids was then calculated for each condition either focusing on the early-stage regulation (Rock Inhibitor, IWR1e, IGF1, SB431542/LDN193189) or late-stage regulation (CHIR99021, SU5402, CHIR99021/SU5402, DAPT) of signaling pathways.

Condition	Number of Retinal Organoids	Number of Non-Retinal Organoids	Yield of Retinal Organoids ^1^
Control	9	80	10.1%
Rock Inhibitor	3	114	2.6%
IGF1	17	78	17.9%
IWR1e	0	42	0.0%
SB431542 + LDN193189	1	106	0.9%
CHIR99021	105	99	51.5%
SU5402	10	118	7.8%
CHIR99021 + SU5402	56	175	24.2%
DAPT	10	112	8.2%

^1^ *n* = 1 for quantification of retinal organoid yields.

## Data Availability

All data generated or analyzed during this study are included in this published article (and its Supplementary Materials files).

## Supplementary Materials

The following are available online at https://www.mdpi.com/article/10.3390/ijms22137081/s1. Table S1. A list of primer sets used for RT-PCR analysis. Figure S1. Brightfield images of all conditions throughout development. Figure S2. Venn diagrams showing the specificity and exclusivity of certain methods and additions.
