# Supplementary material for: The Role of Small Molecules and Their Effect on the Molecular Mechanisms of Early Retinal Organoid Development
Source: Int J Mol Sci. 2021 Jun 30;22(13):7081. doi: 10.3390/ijms22137081 (PMC8268497; doi:10.3390/ijms22137081)
Supplement: Supplementary file 1 [file ijms-22-07081-s001.zip › ijms-1264349-supplementary.pdf]

International Journal of
*Molecular Sciences*

MDPI

*Review*

# The Role of Small Molecules and Their Effect on the Molecular Mechanisms of Early Retinal Organoid Development

**Philip E. Wagstaff [1,*], Andrea Heredero Berzal [2], Camiel J. F. Boon [2,3], Peter M. J. Quinn [4], Anneloor L. M. A. ten Asbroek [1] and Arthur A. Bergen [1,2,5,*]**

[1] Department of Human Genetics, Amsterdam UMC, University of Amsterdam (UvA), 1105 AZ Amsterdam, The Netherlands; a.l.tenasbroek@amsterdamumc.nl
[2] Department of Ophthalmology, Amsterdam UMC, University of Amsterdam (UvA), 1105 AZ Amsterdam, The Netherlands; a.herederoberzal@amsterdamumc.nl (A.H.B.); Camiel.boon@amsterdamumc.nl (C.J.F.B.)
[3] Department of Ophthalmology, Leiden University Medical Center (LUMC), 2333 ZA Leiden,
The Netherlands
[4] Jonas Children's Vision Care and Bernard & Shirlee Brown Glaucoma Laboratory, Columbia Stem Cell Initiative, Departments of Ophthalmology, Pathology & Cell Biology, Institute of Human Nutrition, Vagelos College of Physicians and Surgeons, Columbia University. New York, NY, USA; Edward S. Harkness Eye Institute, Department of Ophthalmology, Columbia University Irving Medical Center—New York-Presbyterian Hospital, New York, NY 10032, USA; pq2138@cumc.columbia.edu
[5] Netherlands Institute for Neuroscience (NIN-KNAW), 1105 BA Amsterdam, The Netherlands
* Correspondence: p.e.wagstaff@amsterdamumc.nl (P.E.W.); aabergen@amsterdamumc.nl (A.A.B.)

# <u>Supplementary Data</u>

| Gene | F/R | Sequence | Product BP |
|---|---|---|---|
| EEF1a | F | CAAAGCGACCCAAAGGTGGAT | 219 |
| | R | AAATAAGCGCCGGCTATGCC | |
| NANOG | F | TTTGGAAGCTGCTGGGGAAG | 194 |
| | R | GATGGGAGGAGGGGAGAGGA | |
| PAX6 | F | AACAGACACAGCCCTCACAAACA | 275 |
| | R | CGGGAACTTGAACTGGAACTGAC | |
| RAX | F | GCGAAACTGTCAGAGGAGGAA | 236 |
| | R | ATGGAGGACACTTCCAGCTTC | |
| VSX2 | F | CCATGCCTAAAGCCCATTGC | 240 |
| | R | TCTGGACTCCACTGATGGGT | |
| MITF | F | TTGCAACGAGAACAGCAACG | 225 |
| | R | ATGCTGAAGGAGGTCTTGGC | |
| ATOH7 | F | CAGCCTGGTCATCCAGTAGAACA | 100 |
| | R | GAGCAAATAAGTCATAAACAAAGCAAC | |

**Supplementary Table S1. Primers used for RT-PCR analysis**

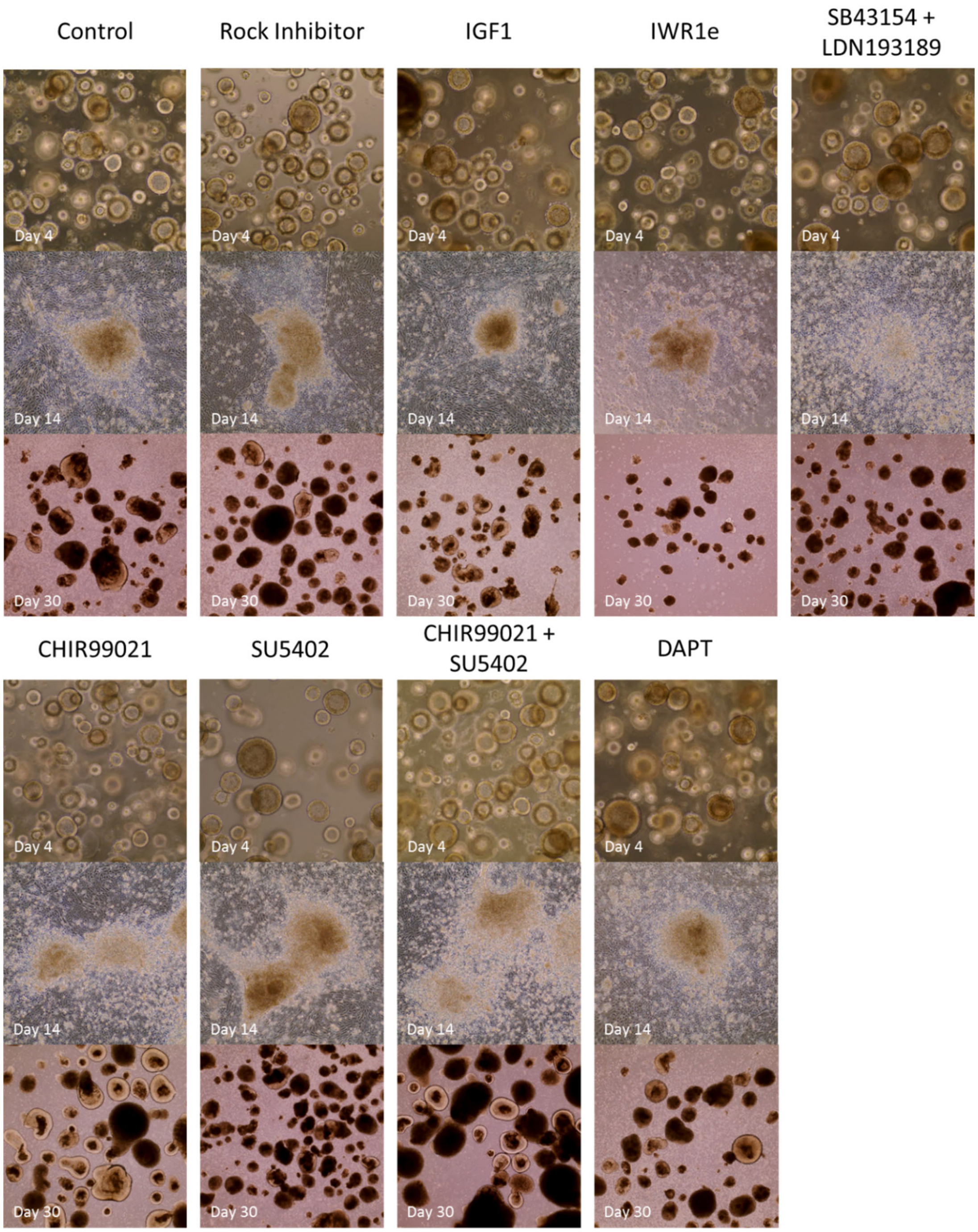


**Figure S1. Retinal organoid development over time was analyzed for all conditions through brightfield microscopy.** From left to right we present the 9 different conditions (Top: Control, Rock Inhibitor, IGF1, IWR1e, SB431542+LDN193189. Bottom: CHIR99021, SU5402, CHIR99021+SU5402, DAPT). These were analyzed by microscopy throughout development. All conditions were comparable by day 4, with some changes in Rock Inhibitor, IWR1e and SB431542+LDN193189 treated cells by day 14. At day 30, the differences in retinal organoid yield were apparent, with some conditions generating more retinal organoids than others.

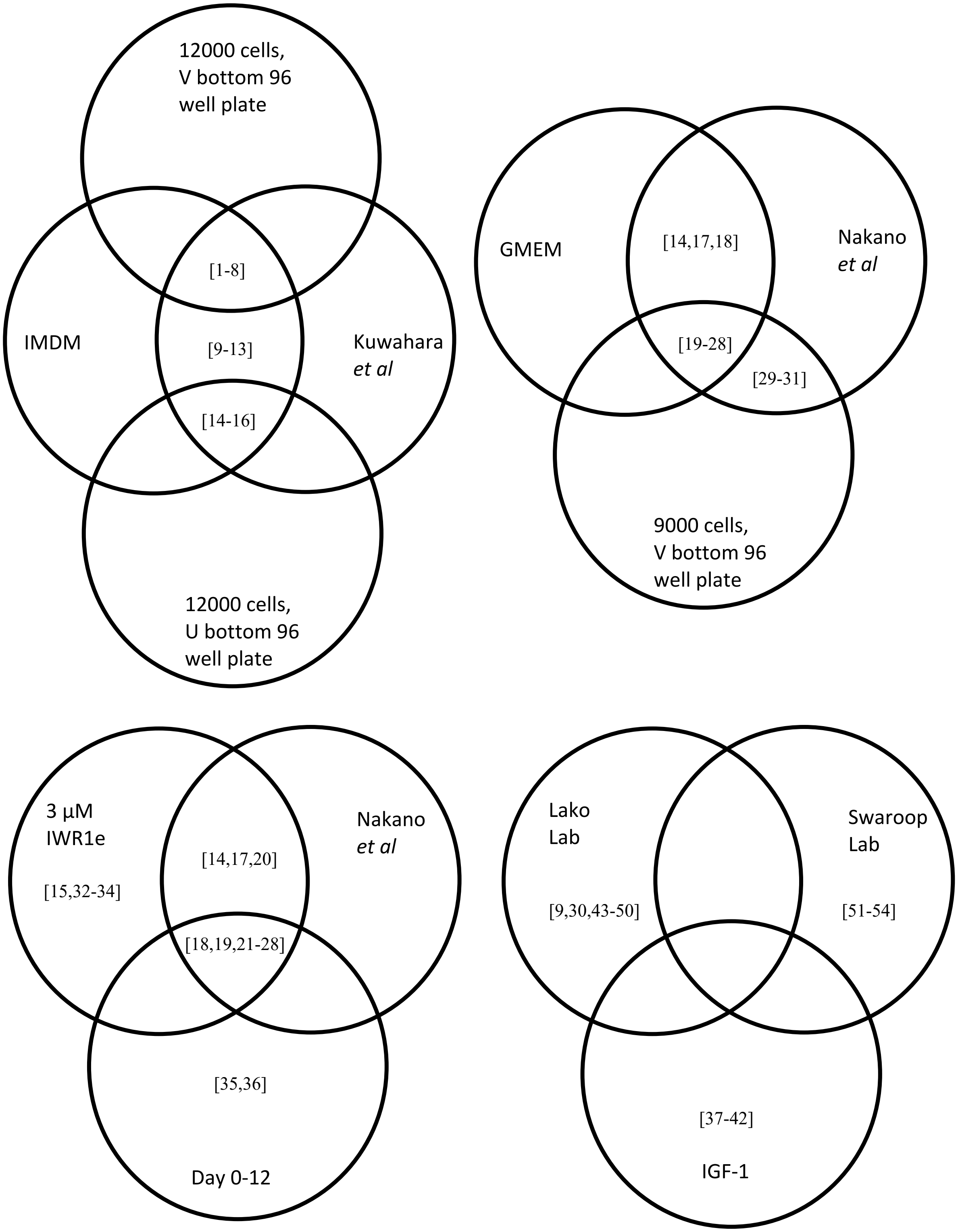
12000 cells,
V bottom 96
well plate
IMDM
Kuwahara
*et al*
12000 cells,
U bottom 96
well plate
[1-8]
[9-13]
[14-16]
GMEM
Nakano
*et al*
9000 cells,
V bottom 96
well plate
[14,17,18]
[19-28]
[29-31]
3 µM
IWR1e
[15,32-34]
Nakano
*et al*
[14,17,20]
[18,19,21-28]
[35,36]
Day 0-12
Lako
Lab
[9,30,43-50]
Swaroop
Lab
[51-54]
[37-42]
IGF-1

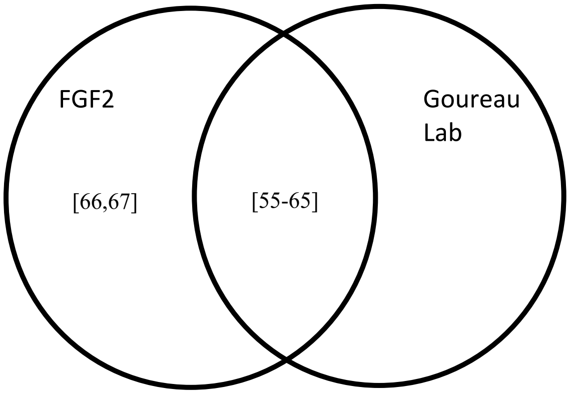


**Figure S2. The specificity of certain methods and additions.** A large variety of types and combinations of culture plates and medium were used in different studies, as indicated by the Venn diagrams above. Some plate-type and additions have been unique to a few studies or laboratories (FGF2 and IGF-1). For example, FGF2 and IGF-1 have been largely used only by the lab of Goureau (FGF2), and the labs of Lako and Swaroop (IGF-1). The use of other specific methods, such as the use of IMDM and GMEM, were introduced by Kuwahara *et al* and by Nakano *et al*. Subsequently, many groups followed-up using the same methods, making them become more wide-spread in the scientific community.

## Supplementary References

1 Deng, W. L. *et al.* Gene Correction Reverses Ciliopathy and Photoreceptor Loss in iPSC-Derived Retinal Organoids from Retinitis Pigmentosa Patients. *Stem Cell Reports* **10**, 1267-1281, doi:10.1016/j.stemcr.2018.02.003 (2018).

2 Guo, Y. *et al.* Modeling Retinitis Pigmentosa: Retinal Organoids Generated From the iPSCs of a Patient With the USH2A Mutation Show Early Developmental Abnormalities. *Front Cell Neurosci* **13**, 361, doi:10.3389/fncel.2019.00361 (2019).

3 Kobayashi, W. *et al.* Culture Systems of Dissociated Mouse and Human Pluripotent Stem Cell-Derived Retinal Ganglion Cells Purified by Two-Step Immunopanning. *Invest Ophthalmol Vis Sci* **59**, 776-787, doi:10.1167/iovs.17-22406 (2018).

4 Kuwahara, A. *et al.* Generation of a ciliary margin-like stem cell niche from self-organizing human retinal tissue. *Nat Commun* **6**, 6286, doi:10.1038/ncomms7286 (2015).

5 Li, Y. P., Deng, W. L. & Jin, Z. B. Modeling retinitis pigmentosa through patient-derived retinal organoids. *STAR Protoc* **2**, 100438, doi:10.1016/j.xpro.2021.100438 (2021).

6 Liu, H. *et al.* Human embryonic stem cell-derived organoid retinoblastoma reveals a cancerous origin. *Proc Natl Acad Sci U S A* **117**, 33628-33638, doi:10.1073/pnas.2011780117 (2020).

7 Liu, H., Hua, Z. Q. & Jin, Z. B. Modeling human retinoblastoma using embryonic stem cell-derived retinal organoids. *STAR Protoc* **2**, 100444, doi:10.1016/j.xpro.2021.100444 (2021).

8 Wang, S., Poli, S., Liang, X. & Peng, G. H. Longitudinal single-cell RNA-seq of hESCs-derived retinal organoids. *Sci China Life Sci*, doi:10.1007/s11427-020-1836-7 (2021).

9 Chichagova, V. *et al.* Human iPSC differentiation to retinal organoids in response to IGF1 and BMP4 activation is line- and method-dependent. *Stem Cells* **38**, 195-201, doi:10.1002/stem.3116 (2020).

10 Georgiou, M. *et al.* Room temperature shipment does not affect the biological activity of pluripotent stem cell-derived retinal organoids. *PLoS One* **15**, e0233860, doi:10.1371/journal.pone.0233860 (2020).

11 Peskova, L. *et al.* miR-183/96/182 cluster is an important morphogenetic factor targeting PAX6 expression in differentiating human retinal organoids. *Stem Cells*, doi:10.1002/stem.3272 (2020).

12 Zeng, Y. *et al.* The Impact of Particulate Matter (PM2.5) on Human Retinal Development in hESC-Derived Retinal Organoids. *Front Cell Dev Biol* **9**, 607341, doi:10.3389/fcell.2021.607341 (2021).

13 Zou, T. *et al.* Organoid-derived C-Kit(+)/SSEA4(-) human retinal progenitor cells promote a protective retinal microenvironment during transplantation in rodents. *Nat Commun* **10**, 1205, doi:10.1038/s41467-019-08961-0 (2019).

14 Browne, A. W. *et al.* Structural and Functional Characterization of Human Stem-Cell-Derived Retinal Organoids by Live Imaging. *Invest Ophthalmol Vis Sci* **58**, 3311-3318, doi:10.1167/iovs.16-20796 (2017).

15 Döpper, H. *et al.* Differentiation Protocol for 3D Retinal Organoids, Immunostaining and Signal Quantitation. *Curr Protoc Stem Cell Biol* **55**, e120, doi:10.1002/cpsc.120 (2020).

16 Hallam, D. *et al.* Human-Induced Pluripotent Stem Cells Generate Light Responsive Retinal Organoids with Variable and Nutrient-Dependent Efficiency. *Stem Cells* **36**, 1535-1551, doi:10.1002/stem.2883 (2018).

17 Aparicio, J. G. *et al.* Temporal expression of CD184(CXCR4) and CD171(L1CAM) identifies distinct early developmental stages of human retinal ganglion cells in embryonic stem cell derived retina. *Exp Eye Res* **154**, 177-189, doi:10.1016/j.exer.2016.11.013 (2017).

18 Gao, M. L. *et al.* Patient-Specific Retinal Organoids Recapitulate Disease Features of Late-Onset Retinitis Pigmentosa. *Front Cell Dev Biol* **8**, 128, doi:10.3389/fcell.2020.00128 (2020).

19 Arno, G. *et al.* Mutations in REEP6 Cause Autosomal-Recessive Retinitis Pigmentosa. *Am J Hum Genet* **99**, 1305-1315, doi:10.1016/j.ajhg.2016.10.008 (2016).

20 Dulla, K. *et al.* Splice-Modulating Oligonucleotide QR-110 Restores CEP290 mRNA and Function in Human c.2991+1655A>G LCA10 Models. *Mol Ther Nucleic Acids* **12**, 730-740, doi:10.1016/j.omtn.2018.07.010 (2018).

21 Eastlake, K. *et al.* Phenotypic and Functional Characterization of Müller Glia Isolated from Induced Pluripotent Stem Cell-Derived Retinal Organoids: Improvement of Retinal Ganglion Cell Function upon Transplantation. *Stem Cells Transl Med* **8**, 775-784, doi:10.1002/sctm.18-0263 (2019).

22 Kaewkhaw, R. *et al.* Transcriptome Dynamics of Developing Photoreceptors in Three-Dimensional Retina Cultures Recapitulates Temporal Sequence of Human Cone and Rod Differentiation Revealing Cell Surface Markers and Gene Networks. *Stem Cells* **33**, 3504-3518, doi:10.1002/stem.2122 (2015).

23 Lane, A. *et al.* Modeling and Rescue of RP2 Retinitis Pigmentosa Using iPSC-Derived Retinal Organoids. *Stem Cell Reports* **15**, 67-79, doi:10.1016/j.stemcr.2020.05.007 (2020).

24 Nakano, T. *et al.* Self-formation of optic cups and storable stratified neural retina from human ESCs. *Cell Stem Cell* **10**, 771-785, doi:10.1016/j.stem.2012.05.009 (2012).

25 Pan, D. *et al.* COCO enhances the efficiency of photoreceptor precursor differentiation in early human embryonic stem cell-derived retinal organoids. *Stem Cell Res Ther* **11**, 366, doi:10.1186/s13287-020-01883-5 (2020).

26 Parfitt, D. A. *et al.* Identification and Correction of Mechanisms Underlying Inherited Blindness in Human iPSC-Derived Optic Cups. *Cell Stem Cell* **18**, 769-781, doi:10.1016/j.stem.2016.03.021 (2016).

27 Völkner, M. *et al.* Retinal Organoids from Pluripotent Stem Cells Efficiently Recapitulate Retinogenesis. *Stem Cell Reports* **6**, 525-538, doi:10.1016/j.stemcr.2016.03.001 (2016).

28 Zheng, C., Schneider, J. W. & Hsieh, J. Role of RB1 in human embryonic stem cell-derived retinal organoids. *Dev Biol* **462**, 197-207, doi:10.1016/j.ydbio.2020.03.011 (2020).

29 Khan, M. *et al.* Detailed Phenotyping and Therapeutic Strategies for Intronic ABCA4 Variants in Stargardt Disease. *Mol Ther Nucleic Acids* **21**, 412-427, doi:10.1016/j.omtn.2020.06.007 (2020).

30 Mellough, C. B. *et al.* Systematic Comparison of Retinal Organoid Differentiation from Human Pluripotent Stem Cells Reveals Stage Specific, Cell Line, and Methodological Differences. *Stem Cells Transl Med* **8**, 694-706, doi:10.1002/sctm.18-0267 (2019).

31 Tornabene, P. *et al.* Intein-mediated protein trans-splicing expands adeno-associated virus transfer capacity in the retina. *Sci Transl Med* **11**, doi:10.1126/scitranslmed.aav4523 (2019).

32 Eldred, K. C. *et al.* Thyroid hormone signaling specifies cone subtypes in human retinal organoids. *Science* **362**, doi:10.1126/science.aau6348 (2018).

33 Lu, Y. *et al.* Single-Cell Analysis of Human Retina Identifies Evolutionarily Conserved and Species-Specific Mechanisms Controlling Development. *Dev Cell* **53**, 473-491.e479, doi:10.1016/j.devcel.2020.04.009 (2020).

34 Wahlin, K. J. *et al.* Photoreceptor Outer Segment-like Structures in Long-Term 3D Retinas from Human Pluripotent Stem Cells. *Sci Rep* **7**, 766, doi:10.1038/s41598-017-00774-9 (2017).

35 Sharma, T. P. *et al.* Patient-specific induced pluripotent stem cells to evaluate the pathophysiology of TRNT1-associated Retinitis pigmentosa. *Stem Cell Res* **21**, 58-70, doi:10.1016/j.scr.2017.03.005 (2017).

36 Wiley, L. A. *et al.* cGMP production of patient-specific iPSCs and photoreceptor precursor cells to treat retinal degenerative blindness. *Sci Rep* **6**, 30742, doi:10.1038/srep30742 (2016).

37 Singh, R. K. *et al.* Characterization of Three-Dimensional Retinal Tissue Derived from Human Embryonic Stem Cells in Adherent Monolayer Cultures. *Stem Cells Dev* **24**, 2778-2795, doi:10.1089/scd.2015.0144 (2015).

38 Singh, R. K., Occelli, L. M., Binette, F., Petersen-Jones, S. M. & Nasonkin, I. O. Transplantation of Human Embryonic Stem Cell-Derived Retinal Tissue in the Subretinal Space of the Cat Eye. *Stem Cells Dev* **28**, 1151-1166, doi:10.1089/scd.2019.0090 (2019).

39 Singh, R. K., Winkler, P. A., Binette, F., Petersen-Jones, S. M. & Nasonkin, I. O. Comparison of Developmental Dynamics in Human Fetal Retina and Human Pluripotent Stem Cell-Derived Retinal Tissue. *Stem Cells Dev* **30**, 399-417, doi:10.1089/scd.2020.0085 (2021).

40 Zhang, X. *et al.* Characterization of CRB1 splicing in retinal organoids derived from a patient with adult-onset rod-cone dystrophy caused by the c.1892A>G and c.2548G>A variants. *Mol Genet Genomic Med* **8**, e1489, doi:10.1002/mgg3.1489 (2020).

41 Zhang, X. *et al.* Gene correction of the CLN3 c.175G>A variant in patient-derived induced pluripotent stem cells prevents pathological changes in retinal organoids. *Mol Genet Genomic Med* **9**, e1601, doi:10.1002/mgg3.1601 (2021).

42 Zhu, J. *et al.* Generation of Transplantable Retinal Photoreceptors from a Current Good Manufacturing Practice-Manufactured Human Induced Pluripotent Stem Cell Line. *Stem Cells Transl Med* **7**, 210-219, doi:10.1002/sctm.17-0205 (2018).

43 Chichagova, V. *et al.* Differentiation of Retinal Organoids from Human Pluripotent Stem Cells. *Curr Protoc Stem Cell Biol* **50**, e95, doi:10.1002/cpsc.95 (2019).

44 Collin, J. *et al.* Deconstructing Retinal Organoids: Single Cell RNA-Seq Reveals the Cellular Components of Human Pluripotent Stem Cell-Derived Retina. *Stem Cells* **37**, 593-598, doi:10.1002/stem.2963 (2019).

45 Collin, J. *et al.* CRX Expression in Pluripotent Stem Cell-Derived Photoreceptors Marks a Transplantable Subpopulation of Early Cones. *Stem Cells* **37**, 609-622, doi:10.1002/stem.2974 (2019).

46 Dorgau, B. *et al.* Decellularised extracellular matrix-derived peptides from neural retina and retinal pigment epithelium enhance the expression of synaptic markers and light responsiveness of human pluripotent stem cell derived retinal organoids. *Biomaterials* **199**, 63-75, doi:10.1016/j.biomaterials.2019.01.028 (2019).

47 Dorgau, B. *et al.* Laminin γ3 plays an important role in retinal lamination, photoreceptor organisation and ganglion cell differentiation. *Cell Death Dis* **9**, 615, doi:10.1038/s41419-018-0648-0 (2018).
48 Felemban, M. *et al.* Extracellular matrix component expression in human pluripotent stem cell-derived retinal organoids recapitulates retinogenesis in vivo and reveals an important role for IMPG1 and CD44 in the development of photoreceptors and interphotoreceptor matrix. *Acta Biomater* **74**, 207-221, doi:10.1016/j.actbio.2018.05.023 (2018).
49 Mellough, C. B. *et al.* IGF-1 Signaling Plays an Important Role in the Formation of Three-Dimensional Laminated Neural Retina and Other Ocular Structures From Human Embryonic Stem Cells. *Stem Cells* **33**, 2416-2430, doi:10.1002/stem.2023 (2015).
50 Zerti, D. *et al.* Developing a simple method to enhance the generation of cone and rod photoreceptors in pluripotent stem cell-derived retinal organoids. *Stem Cells* **38**, 45-51, doi:10.1002/stem.3082 (2020).
51 Kaya, K. D. *et al.* Transcriptome-based molecular staging of human stem cell-derived retinal organoids uncovers accelerated photoreceptor differentiation by 9-cis retinal. *Mol Vis* **25**, 663-678 (2019).
52 Kelley, R. A., Chen, H. Y., Swaroop, A. & Li, T. Accelerated Development of Rod Photoreceptors in Retinal Organoids Derived from Human Pluripotent Stem Cells by Supplementation with 9-cis Retinal. *STAR Protoc* **1**, doi:10.1016/j.xpro.2020.100033 (2020).
53 Kruczek, K. *et al.* Gene Therapy of Dominant CRX-Leber Congenital Amaurosis using Patient Stem Cell-Derived Retinal Organoids. *Stem Cell Reports* **16**, 252-263, doi:10.1016/j.stemcr.2020.12.018 (2021).
54 Regent, F. *et al.* A simple and efficient method for generating human retinal organoids. *Mol Vis* **26**, 97-105 (2020).
55 Gagliardi, G. *et al.* Characterization and Transplantation of CD73-Positive Photoreceptors Isolated from Human iPSC-Derived Retinal Organoids. *Stem Cell Reports* **11**, 665-680, doi:10.1016/j.stemcr.2018.07.005 (2018).
56 Garita-Hernandez, M. *et al.* Control of Microbial Opsin Expression in Stem Cell Derived Cones for Improved Outcomes in Cell Therapy. *Front Cell Neurosci* **15**, 648210, doi:10.3389/fncel.2021.648210 (2021).
57 Garita-Hernandez, M. *et al.* Optogenetic Light Sensors in Human Retinal Organoids. *Front Neurosci* **12**, 789, doi:10.3389/fnins.2018.00789 (2018).
58 Garita-Hernandez, M. *et al.* AAV-Mediated Gene Delivery to 3D Retinal Organoids Derived from Human Induced Pluripotent Stem Cells. *Int J Mol Sci* **21**, doi:10.3390/ijms21030994 (2020).
59 Khabou, H. *et al.* Noninvasive gene delivery to foveal cones for vision restoration. *JCI Insight* **3**, doi:10.1172/jci.insight.96029 (2018).
60 Rabesandratana, O. *et al.* Generation of a Transplantable Population of Human iPSC-Derived Retinal Ganglion Cells. *Front Cell Dev Biol* **8**, 585675, doi:10.3389/fcell.2020.585675 (2020).
61 Reichman, S. *et al.* Generation of Storable Retinal Organoids and Retinal Pigmented Epithelium from Adherent Human iPS Cells in Xeno-Free and Feeder-Free Conditions. *Stem Cells* **35**, 1176-1188, doi:10.1002/stem.2586 (2017).
62 Reichman, S. *et al.* From confluent human iPS cells to self-forming neural retina and retinal pigmented epithelium. *Proc Natl Acad Sci U S A* **111**, 8518-8523, doi:10.1073/pnas.1324212111 (2014).
63 Scholler, J. *et al.* Dynamic full-field optical coherence tomography: 3D live-imaging of retinal organoids. *Light Sci Appl* **9**, 140, doi:10.1038/s41377-020-00375-8 (2020).
64 Slembrouck-Brec, A., Nanteau, C., Sahel, J. A., Goureau, O. & Reichman, S. Defined Xeno-free and Feeder-free Culture Conditions for the Generation of Human iPSC-derived Retinal Cell Models. *J Vis Exp*, doi:10.3791/57795 (2018).

65 Slembrouck-Brec, A. *et al.* Reprogramming of Adult Retinal Müller Glial Cells into Human-Induced Pluripotent Stem Cells as an Efficient Source of Retinal Cells. *Stem Cells Int* **2019**, 7858796, doi:10.1155/2019/7858796 (2019).

66 Diakatou, M. *et al.* Allele-Specific Knockout by CRISPR/Cas to Treat Autosomal Dominant Retinitis Pigmentosa Caused by the G56R Mutation in NR2E3. *Int J Mol Sci* **22**, doi:10.3390/ijms22052607 (2021).

67 Freude, K. K. *et al.* Enrichment of retinal ganglion and Müller glia progenitors from retinal organoids derived from human induced pluripotent stem cells - possibilities and current limitations. *World J Stem Cells* **12**, 1171-1183, doi:10.4252/wjsc.v12.i10.1171 (2020).
